# Impact of Center Volume on Cardiopulmonary and Mortality Outcomes after Immune-Checkpoint Inhibitors for Cancer: A Systematic Review and Meta-Analysis

**DOI:** 10.3390/cancers16061136

**Published:** 2024-03-13

**Authors:** Mohamed Rahouma, Nathan Mynard, Massimo Baudo, Sherif Khairallah, Shaikha Al-Thani, Anas Dabsha, Shon Shmushkevich, Osama Shoeib, Mohamed Hossny, Elsayed Eldeeb, Hala Aziz, Naglaa Abdelkarim, Mario Gaudino, Abdelrahman Mohamed, Leonard Girardi, Jun Zhang, Luciano Mutti

**Affiliations:** 1 Department of Cardiothoracic Surgery, Weill Cornell Medicine, New York, NY 10065, USA; jam9306@nyp.org (N.M.); m.baudo@unibs.it (M.B.); smk4005@med.cornell.edu (S.K.); sma9023@nyp.org (S.A.-T.); dabshaa@nychhc.org (A.D.); sshmu001@med.fiu.edu (S.S.); mohamedheshamhosny@outlook.com (M.H.); mfg9004@med.cornell.edu (M.G.); lngirard@med.cornell.edu (L.G.); 2 Surgical Oncology Department, National Cancer Institute, Cairo University, Cairo 11796, Egypt; rahmannci@yahoo.com; 3 Department of Cardiac Surgery, Spedali Civili di Brescia, 25123 Brescia, Italy; 4 Department of Cardiology, Tanta University, Tanta 31512, Egypt; oshouip@gmail.com; 5 E H Reliable Medical PC, Lutheran NYU Langone, New York, NY 11214, USA; samalexandria74@gmail.com; 6 Medical Oncology Department, National Cancer Institute, Cairo University, Cairo 11796, Egypt; halaaziz2001@yahoo.com; 7 Department of Medicine, Hematology and Oncology, Medical College of Georgia, Augusta University, Augusta, GA 30912, USA; nkarim@augusta.edu; 8 Division of Medical Oncology, Department of Internal Medicine, University of Kansas Medical Center, Kansas City, KS 66103, USA; jzhang3@kumc.edu; 9 Department of Cancer Biology, University of Kansas Cancer Center, Kansas City, KS 66103, USA; 10 Center for Biotechnology, Sbarro Institute for Cancer Research and Molecular Medicine, College of Science and Technology, Temple University, Philadelphia, PA 19122, USA; luciano.mutti@temple.edu; 11 Department of Applied Clinical Sciences and Biotechnology, L’Aquila University, 67100 L’Aquila, Italy

**Keywords:** adverse events, immunotherapy, checkpoint inhibitors, meta-analysis, cancer, hospital volume

## Abstract

**Simple Summary:**

Immune-checkpoint inhibitors have been proven to aid in tumor regression. We performed a meta-analysis and systematic review to investigate the relationship between hospital volume and patient outcomes. We found that centers performing ≥ 33 annual cases had fewer cardiac-related adverse events (AEs). The pooled estimated rates of grade 5 AEs were 2.75% (95%CI: 2.18–3.47), with higher annual cases associated with reduced grade 5 AEs at meta-regression. Ultimately, patients should be referred to high-volume centers when severe immune-related AEs occur.

**Abstract:**

Immune-checkpoint inhibitors (ICIs) were proven effective in inducing tumor regression. However, its toxicity tends to be fatal. We sought to investigate the hospital volume/outcomes relationship. Databases were searched for studies reporting immune-checkpoint inhibitors adverse events (AEs) in patients with solid-organ malignancies. The outcomes were A) the pooled events rate (PER) of grade 5, grade 3–4, cardiac-related, and pulmonary-related AEs, and B) the assessment of the volume/outcomes relationship. One hundred and forty-seven studies met our inclusion criteria. The PER of grade 5, grade 3–4, and any-grade AEs was 2.75% (95%CI: 2.18–3.47), 26.69% (95%CI: 21.60–32.48), and 77.80% (95%CI: 70.91–83.44), respectively. The PER of pulmonary-related AEs was 4.56% (95%CI: 3.76–5.53). A higher number of annual cases per center was significantly associated with reduced grade 5 (*p* = 0.019), grade 3–4 (*p* = 0.004), and cardiac-related AEs (*p* = 0.035) in the meta-regression. In the current era of cancer immunotherapy, knowledge regarding the early diagnosis and management of immunotherapy-related AEs is essential. Our meta-analysis demonstrates the importance of center volume in improving outcomes and reducing the incidence of severe AEs.

## 1. Introduction

Our immune system plays a pivotal role in cancer prevention and defense. Cytotoxic T lymphocytes (CD8+) and natural killer cells are the effector immune cells that destroy cancer cells directly by the induction of apoptosis and indirectly by the production of cytokines that activate other immune cells against cancer [1,2,3,4,5]. Cancer cells always try to evade the immune response by overexpressing PD (programmed death) receptors on the surface of the effector immune cells and PD-Ligand (PDL) on its surface, and the interaction between the ligand and the receptor inhibits the T-cells from initiating an immune response against cancer [6,7,8]. Anti-PD-1, anti-PDL1, and anti-CTLA4 (Cytotoxic T-Lymphocyte Associated Protein 4) are monoclonal antibodies against these ligands and receptors and proved to be effective in inducing tumor regression in different tumors, especially when combined with other traditional anti-cancer drugs [9,10,11,12,13,14]. However, the emergence of new immune-related serious adverse events (irAEs) is quite common and occasionally can be severe enough to cause mortality [15,16,17,18]. This, combined with the financial impact of these therapies, creates the need for high-volume centers and well-trained medical teams to improve the outcomes of these drugs. Hospital volume/outcome relationships have been extensively studied before for cancer [19,20,21,22] and non-cancer-related issues [23,24,25]. A paper analyzing patients undergoing chemotherapy for acute myeloid leukemia showed that higher-volume hospitals reported lower mortality rates compared to low-volume ones. A possible explanation may be that more experienced house staff can recognize and manage chemotherapy complications at an earlier stage, leading to fewer deaths [26]. Most experts recommend against immunotherapy administration in low-volume centers [27]. No meta-analysis has addressed this issue related to immune-checkpoint inhibitors so far. We sought to investigate this issue on immune-checkpoint inhibitors with a meta-analysis of existing data, including only the prospective and single-center studies, to accurately assess the volume/outcomes relationship by an annual number of patients.

## 2. Materials and Methods

### 2.1. Search Strategy and Study Selection

The present meta-analysis was conducted according to the Preferred Reporting Items for Systematic Reviews and Meta-Analyses (PRISMA) guidelines (File S1: PRISMA checklist) [28]. Ovid MEDLINE, Ovid Embase, PubMed, and Cochrane Library were searched for publications in the English language up to February 2022 with no date restriction. Search terms included all subject headings and associated keywords for “immunotherapy”, “PD-1 inhibitor”, “PD-L1 inhibitor”, “CTLA-4 inhibitor” and “cancer”. Reference lists of included papers were also searched for potential inclusion of eligible studies (i.e., backward snowballing). The main objective of this meta-analysis is to evaluate the relation between the hospital volume (the number of cases per year), which reflects the clinical experience, and the incidence rate of irAEs. The inclusion criteria were as follows: single-center prospective (either randomized or not) studies, so as to evaluate annual hospital volume; eligible studies must have addressed irAEs in patients with solid-organ malignancies only; and hematological ones were excluded due to different pathophysiology. All studies with at least one arm containing anti-PD-1 or anti-PDL-1 or CTLA-4 agents (with or without other non-immunotherapy treatments) were included. We excluded other publications, including non-English studies, review articles, retrospective studies, multicenter studies, case reports/series, editorials, guidelines, and abstracts. In studies with overlapping populations, we included the study with the largest sample size.

After de-duplicating of search results, four medically qualified reviewers (M.B., N.M., A.D., S.K.) screened 1574 citations. A fifth independent reviewer (M.R.) confirmed adequacy of studies based on predefined inclusion and exclusion criteria for titles and abstracts.

Full-text articles of initially screened titles and abstracts were then retrieved for a second round of eligibility screening (PRISMA flow chart shown in Supplementary Figure S1).

This review was registered with the PROSPERO Register of Systematic Reviews (CRD42021285674). No individual patients were involved in this study, so institutional review board approval was not required.

### 2.2. Data Extraction and Quality Assessment

Five investigators (A.D., M.H., O.S., N.M., S.K.) independently extracted data. All the following data were retrieved for each study: study characteristics (the author, publication year, study period, and institute), patients’ data (mean age, male percent, associated comorbidities, performance status, and body mass index), tumor pathology and stage, concurrent treatment (surgery, chemotherapy, and radiotherapy), immune-checkpoint inhibitor drug used, any-grade irAEs, grade 3–4 irAEs, grade 5 irAEs (treatment-related mortality), cardio-pulmonary irAEs, mean follow-up, and overall survival.

For any-grade irAEs, we included data from studies that mentioned “any-grade” as a separate variable rather than simple summation of data from different grades as this would lead to overlap of patients and may exceed the 100%; we included all types of cardiac- and pulmonary-related events. Pulmonary complications included pneumonitis, pneumonia, respiratory failure, pleural effusion, pneumothorax, and pleural infection. Cardiac complications included arrhythmias such as ventricular tachycardia and atrial fibrillation, pericardial effusion, pericarditis, myocardial infarction, cardiac myositis, cardiomyopathy, and death from cardiac arrest or acute heart failure. The quality of included studies was assessed using the Cochrane Collaboration’s tool for assessing risk of bias in randomized trials and the Newcastle–Ottawa Quality Assessment Scale (NOS) for critical appraisal for randomized controlled trials (RCTs) and observational studies, respectively [29,30]. Only RCTs and high-quality observational articles (defined as those with a NOS score of 7 or more) were included.

### 2.3. Outcomes

The outcomes of interest were as follows:

(A) The incidence of grade 5 irAEs, defined as treatment-related mortality, grade 3–4 irAEs, any irAEs, cardiac-related irAEs, and pulmonary-related irAEs among the entire cohort.
(B) The incidence of grade 5 irAEs and grade 3–4 irAEs by immune-checkpoint inhibitor (a) name and (b) class, categorized as anti-PD-1, anti-PD-L1, and anti-CTLA-4.
(C) Assessment of volume/outcomes relationship. Volume was defined as annual cases treated with ICIs per center.

Grade severity was reported as defined by the National Cancer Institute Common Terminology Criteria for Adverse Events (NCI-CTCAEv.5). Grade 1: Mild; asymptomatic or mild symptoms; clinical or diagnostic observations only; intervention not indicated. Grade 2: Moderate; minimal, local, or noninvasive intervention indicated; limiting age-appropriate instrumental activities of daily living. Grade 3: Severe or medically significant but not immediately life-threatening; hospitalization or prolongation of hospitalization indicated; disabling; limiting self-care activities of daily living. Grade 4: Life-threatening consequences; urgent intervention indicated. Grade 5: Death related to AEs.

### 2.4. Data Synthesis and Statistical Analysis

Categorical variables were expressed as frequency, while continuous ones were expressed as mean with standard deviation. The annual hospital volume per study was derived by dividing the total number of included patients by the study period, expressed in years.

The pooled event rates (PERs) with a 95% confidence interval [CI] were calculated by weighing the studies with the inverse of the variance of the estimate for that study. The between-study variance was estimated using the Der Simonian–Laird method with the random effects model.

Hypothesis testing for equivalence was set at the two-tailed 0.05 level. Heterogeneity was based on the Cochran Q-test, with I2 values. In the case of significant heterogeneity, defined as I2 > 50%, individual study inference analysis was performed through a “leave-one-out” sensitivity analysis.

Funnel plots by graphical inspection were used for the assessment of publication bias.

The outcomes were further evaluated through subgroup analysis by the class of immune-checkpoint inhibitor and the individual immune-checkpoint inhibitor.

Meta-regression was used to assess the effect of baseline characteristics on the PER of all outcomes. Meta-regression was reported as regression coefficient (beta) ± standard error (SE). A positive beta value corresponds to a higher incidence of the outcome with a higher incidence of the assessed variable. In comparison, a negative beta corresponds to a lower incidence of the outcome with a higher incidence of the assessed variable.

Threshold analysis was performed by testing progressive cut-off values of annual cases on the outcome and looking for a change in significance between the two subgroups (interaction *p*-value). A restricted cubic spline model was used to assess the relationship between the variables.

All analyses were performed using R, version 4.1.0 (R Project for Statistical Computing, Vienna, Austria) and R Studio version 1.4.1717, using the “meta” and “metafor” packages.

## 3. Results

### 3.1. Characteristics of Eligible Studies

We identified a total of 2146 studies in the databases. After the exclusion of duplicates, we screened 1574 studies. A total of 1413 studies were not eligible and were thus excluded. Then, 161 full-text articles were left to be assessed for eligibility. Finally, 147 studies with a total of 4940 patients met the eligibility criteria. Among the included studies, the sample size mean was 27 (IQR: 17–40). The PRISMA flowchart is shown in Supplementary Figure S1. Supplementary Table S1 shows the search terms and strategy used. Supplementary Table S3 shows the studies characteristics and demographics of the included patients. The patients’ average age was 59.22 ± 8.44 years, and men accounted for 47.64% of the included patients, Supplementary Table S4. The included papers comprised 15 (10.2%) RCTs and 132 prospective studies among the included papers. A total of 87 papers were published from the United States, with 14 from China and 9 from Japan. The most frequent cancers were lung (*n* = 29), melanoma (*n* = 24), pancreatic (*n* = 13), and breast (*n* = 11), while adenocarcinoma (*n* = 28) and squamous cell carcinoma (*n* = 19) were the most frequent histology. Further details are depicted in Table 1. Supplementary Table S2 displays the overall quality of the included studies. For the 132 non-RCTs, 102 had a NOS score of 7/9, 28 had a score of 8/9, and 2 had a score of 9/9.

### 3.2. Meta-Analysis

**Figure 1:**
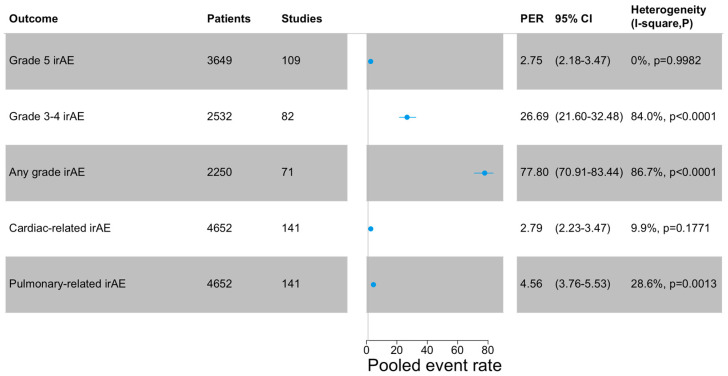
Meta-analysis of the outcomes. Cardiac-related irAEs include pericarditis, heart failure, pericardial fluid, myocarditis, atrial fibrillation. Pulmonary-related irAEs include pneumonitis, dyspnea, pneumonitis, respiratory failure, pleural effusion, and pleural infection. PER: pooled events rate.

The PER of grade 5 irAEs, defined as treatment-related mortality, was 2.75% (95%CI: 2.18–3.47), while for grade 3–4 irAEs, the PER was 26.69% (95%CI: 21.60–32.48). The PER of irAEs (any grade) was 77.80% (95%CI: 70.91–83.44). Cardiac-related irAEs (any grade) reported a PER of 2.79% (95%CI: 2.23–3.47), while for pulmonary-related irAEs (any grade), the PER was 4.56% (95%CI: 3.76–5.53). The outcomes are summarized in Figure 1. A leave-one-out analysis showed the robustness of the obtained estimate. Funnel plots are shown in Supplementary Figure S3.

Further sensitivity analysis was performed by excluding 33 studies that used agents not routinely used in clinical practice and 12 studies that used other molecular inhibitory drugs (Supplementary Table S5). The primary outcomes obtained by excluding those 33 studies were as follows: any-grade AEs: 75.99% (95%CI: 68.12–82.42); grade 3–4 AEs: 24.03% (95%CI: 18.69–30.32); grade 5 AEs: 2.31% (95%CI: 1.77–3.02); cardiac-related AEs: 2.48% (95%CI: 1.88–3.26); pulmonary-related AEs: 4.70% (3.76–5.88). By excluding all 45 studies, the primary outcomes were as follows: any-grade AEs: 77.73% (95%CI: 69.11–84.48); grade 3–4 AEs: 24.69% (95%CI: 18.81–31.69); grade 5 AEs: 2.44% (95%CI: 1.83–3.26); cardiac-related AEs: 2.54% (95%CI: 1.88–3.43); pulmonary-related AEs: 4.51% (3.54–5.75).

### 3.3. Subgroup Analyses and Meta-Regression

In the subgroup analysis on treatment-related mortality (grade 5), no significant subgroup differences were seen by the class of immune-checkpoint inhibitors (*p* = 0.481) and individual drugs (*p* = 0.980) (Supplementary Tables S6 and S7). On the other hand, grade 3–4 irAEs showed a higher trend for CTLA-4 inhibitors (*p* = 0.061) compared to PD-1 and PD-L1 inhibitors. No significant subgroup difference was noted for grade 3–4 irAEs by individual drugs (*p* = 0.116). However, there was a significant difference between pembrolizumab and ipilimumab (*p* = 0.003) (see Supplementary Tables S6 and S7).

A subgroup analysis of the primary outcomes was performed comparing lung cancer-only studies vs. pancreatic cancer-only studies vs. all other cancers (excluding studies reporting a mix of lung and/or pancreatic cancer). Pancreatic cancer only showed significantly higher 3–4 and any-grade irAEs than lung cancer only (sub-group difference *p*-value 0.0113 and 0.0207, respectively). Lung cancer only reported significantly higher pulmonary-related irAEs than pancreatic cancer (subgroup difference *p*-value = 0.0059) (Supplementary Table S8).

**Figure 2:**
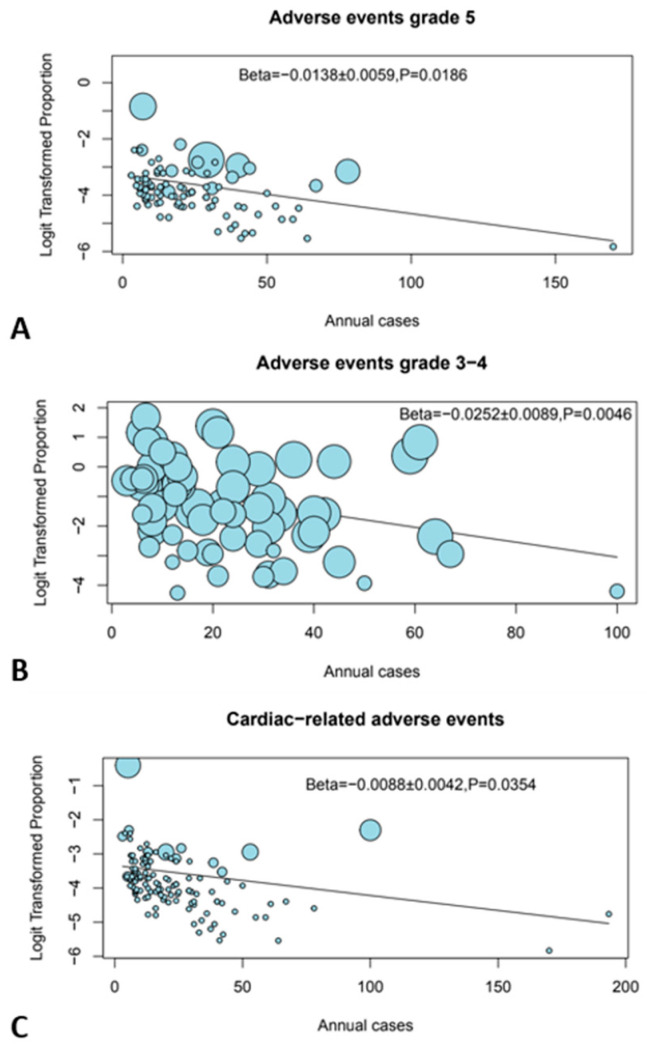
Bubble plots showing the effect of center volume. (**A**) Grade 5 AEs, (**B**) grade 3–4 AEs, and (**C**) cardiac-related AEs.

A higher number of annual cases per center was significantly associated with reduced incidence of grade 3–4, grade 5, and cardiac-related irAEs (*p* = 0.005, *p* = 0.019, and *p* = 0.035, respectively) in the meta-regression (Figure 2). The longer study period of the included papers was significantly associated with fewer grade 5 irAEs (*p* = 0.029), which may reflect the impact of learning curve.

Chemotherapy or surgery was significantly associated with more cardiac-related irAEs (*p* < 0.001). Performance status 1 was significantly and positively associated with more cardiac-related irAEs (*p* = 0.0485). The meta-regression results are summarized in Table 2 and Supplementary Figure S2.

A further analysis was performed through a meta-regression of the additional treatments to the immune-checkpoint inhibitor. Only chemoradiation was significantly associated with cardiac-related adverse events, while all other treatments did not influence the outcomes (Table 3).

The threshold analysis, excluding the 33 papers that used agents not routinely used in clinical practice, revealed that centers performing ≥ 33 annual cases had fewer cardiac-related irAEs when compared to centers performing < 33 annual cases (PER 1.79% [95%CI 1.12–2.85] versus 3.38% [95%CI 2.49–4.56], respectively), *p*-value for interaction = 0.025 Centers that performed ≥ 38 annual cases had fewer pulmonary-related irAEs when compared to centers that performed < 38 annual cases (PER 3.21% [95%CI 1.82–5.59] versus 5.99% [95%CI 4.71–7.57], respectively), *p*-value for interaction = 0.043. Centers performing ≥ 31 annual cases had fewer grade 3–4 irAEs when compared to centers performing < 31 annual cases (PER 12.48% [95%CI 6.44–22.81] versus 27.90% [95%CI 21.28–35.65], respectively), *p*-value for interaction = 0.016. The threshold analysis is summarized in Supplementary Figure S4.

## 4. Discussion

To our knowledge, this is the first meta-analysis reporting on the hospital volume/outcomes relationship in immunotherapy. Interestingly, this meta-analysis met its target by providing evidence for significant correlation: the higher the number of cases treated per year, the lower the incidence of severe AEs from immune-checkpoint inhibitors.

This meta-analysis showed that the higher the annual rate of cases, the lower the incidence of grades 3, 4, and 5 and cardiac-related serious irAEs (hospital volume/outcomes relationship). While pembrolizumab and ipilimumab, the most used drugs in the included studies, appear to have a similar incidence rate of grade 5 irAEs, ipilimumab was associated with greater incidence of grade 3–4 irAEs as compared to pembrolizumab. Patients on immune-checkpoint inhibitors that underwent surgery or received additional chemotherapy or chemoradiation therapy were associated with an increased incidence of cardiac-related severe irAEs.

ICIs are arguably the most important agents for cancer therapy in the current era. Many recent RCTs and meta-analyses have demonstrated their remarkable association with high rates of durable clinical response in patients across a wide range of tumors, including lung [12,14], head and neck [10], glioma [9], colorectal [13], and melanoma [11]. A recent meta-analysis reported improved survival even in cancer patients with marginal performance status [31]. Due to this frequent use, a plethora of irAEs that were distinct from the classical chemotherapy-related toxicities have been frequently encountered. These irAEs can affect different organs and systems, including skin, gastrointestinal, hepatic, cardiovascular, neurological, ocular, thyroid, bone and joints, hematological, and renal systems [27,32,33]. Hence, it became necessary to understand the underlying mechanism of these events to guide the clinical use of these drugs. Early recognition and proper management of these events is vital as these irAEs can be life-threatening if left noticed and untreated [34,35].

Different studies compared the toxicity profile and safety of different classes of immunotherapy and their combination with other targeted and chemotherapy drugs in different cancer types. Most of them concluded that these combinations, though possibly leading to a reduction in the risk of death and tumor progression, and a dramatic increase in the tumor response, could also result in new toxic reactions and more severe irAEs. The authors of [15,16,17,18,36,37,38] reported a higher incidence rate of GIT toxicity with ipilimumab and arthralgia (12%) with pembrolizumab. Xu et al. [38] reported a higher incidence rate of severe irAEs with ipilimumab vs. pembrolizumab (28.6% vs. 19.8%). De Velasco et al. [37], in their meta-analysis, reported a higher risk of all-grade rash (3.94 vs. 1.59, *p* = 0.006) and high-grade colitis (22.5 vs. 2.47, *p* = 0.021) with ipilimumab as compared to PD-1/PD-L1 drugs. They also reported that ipilimumab-induced colitis was the most common cause of irAE-related mortality. Our results are in line with the previously mentioned results.

This study reached its endpoints and gave a conclusive result on the importance of hospital volume on the outcomes for those who were treated with immune-checkpoint inhibitors. This is particularly true for grade 5, grade 3–4, and cardiac-related irAEs; further studies are warranted to investigate this aspect. At the threshold analysis between several annual cases and cardiac-related AEs, 33 annual cases were the limit between high- and low-volume centers, while it was 31 for grade 3–4 AEs and 38 for pulmonary-related AEs.

A possible explanation for the discrepancy between cardiac- and pulmonary-related AEs may be related to the fact that cardiac-related irAEs had fewer low-grade complications compared to pulmonary irAEs (2.48% vs. 14.09%, respectively). With the emergence of new irAEs, it is highly recommended that cancer patients receive these medications in high-volume centers from well-trained medical teams with good knowledge about potential risk factors, surveillance strategies, and critical points in managing patients with immunotherapy irAEs. Over the past two decades, numerous studies and meta-analyses have highlighted the positive impact of high-volume centers in decreasing the morbidity and mortality risks after multidisciplinary management of different types and sites of cancer [19,20,21,22,39,40,41,42,43,44]. In a recent propensity-matched study of pancreatic cancer patients, Hue et al. reported improved overall survival for those treated in at high-volume centers (36.3 vs. 29.4 months; *p* = 0.03; hazard ratio 0.73) [45].

Interestingly, the longer study period of the included papers was associated with lower treatment-related mortality (grade 5 AEs, *p* = 0.0294), which may indirectly reflect the learning curve in early recognition of treatment-related toxicity with consequent early treatment.

Finally, recent technological advancements and the successful clinical implementation of CAR-T cells have broadened the potential applications of adoptive cell therapy, particularly in treating patients with malignant B-cell neoplasms and have now extended to solid tumors [46].

### Strengths and Limitations

Our study had some limitations, as the included studies have some heterogenicity regarding the cancer types and the second treatment arm. However, to make it more homogenous, we performed several subgroup analyses. In addition, different additional agents used concomitantly to the immune-checkpoint inhibitors in certain studies could add toxicities, which may become a possible source of bias. We recalculated the primary outcome by excluding 33 studies that included agents not routinely used in clinical practice. In addition, most of the included papers were phase I or II studies, thus considered high-volume centers and a possible source of bias. Nevertheless, we found significant variability of center volume from this subset of papers ranging from 2 to 170 patients/year; however, we were able to determine the cutoff for high- vs. low-volume centers through the threshold analysis.

Furthermore, we found that the “all grade” cardiac-related irAEs were highly skewed by the high percentage of high-grade AEs, as evidenced by obtaining a mean and a median of 2.45% and 0% of low-grade AEs respectively, compared to pulmonary AEs. This was calculated through the formula in each study: “low-grade cardiac irAEs divided by all grade cardiac irAEs times 100”, and then the mean and median of all the studies were obtained.

Nevertheless, this meta-analysis also has many strengths. For example, it is the first meta-analysis reporting hospital volume/outcomes relationship in immune-checkpoint inhibitors. In addition, the prospective (randomized and non-randomized) and single-center (excluding multi-center studies) nature of the included studies represents an inherited strength by reducing the risk of potential confounding bias on annual hospital volume (low vs. high).

## 5. Conclusions

In the current era, the rapid evolution of cancer immunotherapy will continue to reshape the therapeutic landscape in the coming years, and physicians will increasingly be confronted with common but also rare irAEs. Hence, the knowledge needs to be updated regarding these toxicities’ clinical presentation, diagnosis, and management. Our meta-analysis represents the importance of center volume in improving the outcomes and reducing the incidence of such severe irAEs, based on which we highly recommend immune-checkpoint inhibitors to be administered at a high-volume healthcare center, or its physicians to be consulted and referred to when serious irAEs happen in low-volume centers.

## Figures and Tables

**Table 1:** Baseline patients’ demographic of the included studies.

	Number of Studies (%) (*n* = 147 Studies)
Study type	
RCT	15 (10.2%)
Prospective	132 (89.8%)
Age (Mean ± SD, Median (range))	59.22 ± 8.44, 60.75 (19.00–72.00)
Sex	
Males	70 (47.6%)
Females	77 (52.4%)
Site	
Lung	29 (19.7%)
Melanoma	24 (16.3%)
Pancreas	13 (8.8%)
Breast	11 (7.5%)
Others	70 (47.6%)
Histology	
Adenocarcinoma	28 (19.0%)
Squamous cell carcinoma	19 (12.9%)
Germ cell tumor	2 (1.4%)
Others	98 (66.7%)
Country and region	
United States of America	87 (59.2%)
China	14 (9.5%)
Japan	9 (6.1%)
Germany	6 (4.1%)
Italy	6 (4.1%)
The Netherlands	6 (4.1%)
Belgium	3 (2.0%)
Australia	2 (1.4%)
Canada	2 (1.4%)
Norway	2 (1.4%)
United Kingdom	2 (1.4%)
Denmark	1 (0.7%)
France	1 (0.7%)
Israel	1 (0.7%)
Korea	1 (0.7%)
Mexico	1 (0.7%)
Slovakia	1 (0.7%)
Switzerland	1 (0.7%)
Taiwan	1 (0.7%)

SD = standard deviation.

**Table 2:** Meta-regression of annual cases on each of the outcome.

	Grade 5 irAEs	Grade 3–4 irAEs	Any-Grade irAEs	Cardiac-Related irAEs	Pulmonary-Related irAEs
Variables	Beta ^a^ ± SE, *p*-Value	(Beta ± SE, *p*-Value)	(Beta ± SE, *p*-Value)	(Beta ± SE, *p*-Value)	(Beta ± SE, *p*-Value)
Annual cases	**−0.0138 ± 0.0059, 0.0186**	**−0.0252 ± 0.0089, 0.0046**	−0.0084 ± 0.0116, 0.4718	**−0.0088 ± 0.0042, 0.0354**	−0.0032 ± 0.0032, 0.3151
Study period (years)	**−0.2443 ± 0.1122, 0.0294**	0.0695 ± 0.1317, 0.5976	0.0724 ± 0.1720, 0.6740	−0.0378 ± 0.0936, 0.6859	0.0540 ± 0.0720, 0.4531
Mean Age	−0.0006 ± 0.0157, 0.9699	−0.0039 ± 0.0159, 0.8046	0.0103 ± 0.0205, 0.6167	−0.0238 ± 0.0155, 0.1233	−0.0112 ± 0.0112, 0.3168
Male (%)	−0.0035 ± 0.0043, 0.4099	−0.0012 ± 0.0055, 0.8295	0.0068 ± 0.0070, 0.3282	−0.0056 ± 0.0043, 0.1950	0.0051 ± 0.0039, 0.1925
Performance status 1 (%)	0.0006 ± 0.0059, 0.9196	−0.0065 ± 0.0073, 0.3761	−0.0100 ± 0.0091, 0.2723	**0.0105 ± 0.0053, 0.0485**	−0.0007 ± 0.0041, 0.8589
Underwent surgery (%)	0.0056 ± 0.0038, 0.1412	0.0024 ± 0.0041, 0.5607	−0.0052 ± 0.0060, 0.3843	**0.0166 ± 0.0031, <0.0001**	**−0.0107 ± 0.0034, 0.0019**
Underwent radiotherapy (%)	0.0000 ± 0.0040, 0.9968	0.0004 ± 0.0043, 0.9290	0.0003 ± 0.0059, 0.9602	−0.0009 ± 0.0038, 0.8082	0.0027 ± 0.0034, 0.4367
Underwent chemotherapy (%)	0.0044 ± 0.0034, 0.1961	0.0050 ± 0.0040, 0.2123	0.0071 ± 0.0053, 0.1797	**0.0139 ± 0.0030, <0.0001**	0.0041 ± 0.0031, 0.1938

Values are expressed as: beta ± SE, *p*-value. AEs: adverse events. The bold text represents significant values. ^a^ Beta is the regression coefficient; the higher the significant positive beta, the higher the occurrence of the outcome of interest with a higher occurrence of the variable, while the higher significant negative beta, the lower the occurrence of the outcome of interest with a higher occurrence of the variable.

**Table 3:** Meta-regression of the effect of additional treatments to immune-checkpoint inhibitors on the adverse event outcomes.

	All-Grade irAEs	Grade 3–4 irAEs	Grade 5 irAEs	Cardiac irAEs	Pulmonary irAEs
Treatment	Beta ^a^ ± SE, *p*-Value	(Beta ± SE, *p*-Value)	(Beta ± SE, *p*-Value)	(Beta ± SE, *p*-Value)	(Beta ± SE, *p*-Value)
IO + RTH	−0.4356 ± 0.3760, 0.2466	−0.0176 ± 0.2878, 0.9513	−0.1835 ± 0.2464, 0.4566	0.1976 ± 0.2303, 0.3907	−0.0530 ± 0.2088, 0.7996
IO + CTH	−0.1481 ± 0.3835, 0.6995	0.0097 ± 0.2984, 0.9740	0.3808 ± 0.2557, 0.1365	0.0615 ± 0.2371, 0.7952	0.0143 ± 0.2092, 0.9455
IO + RTH + CTH	−0.6608 ± 0.3941, 0.0936	0.1972 ± 0.3067, 0.5203	0.0818 ± 0.2568, 0.7500	**0.5371 ± 0.2323, 0.0208**	0.0025 ± 0.2279, 0.9911
IO + other	0.7727 ± 0.7813, 0.3226	−0.0501 ± 0.5760, 0.9307	0.3849 ± 0.3366, 0.2528	−0.1529 ± 0.3733, 0.6822	−0.2627 ± 0.3422, 0.4427

CTH: chemotherapy; IO: immunotherapy; RTH: radiotherapy. The bold text represents significant values. ^a^ Beta: is the regression coefficient; the higher significant positive beta, the higher the occurrence of the outcome of interest with a higher occurrence of the variable, while the higher significant negative beta, the lower the occurrence of the outcome of interest with a higher occurrence of the variable.

## Data Availability

The data that support the findings of this study are available from the corresponding author upon reasonable request.

## Supplementary Materials

The following supporting information can be downloaded at: https://www.mdpi.com/article/10.3390/cancers16061136/s1, Figure S1: PRISMA flowchart; Figure S2: Bubble plots showing meta-regression of different variables on pooled events rates; Figure S3: Funnel plots for assessment of publication bias; Figure S4: Threshold analysis showing relation between number of annual cases; Table S1: Search terms used in our meta-analysis; Table S2: Quality of included studies; Table S3: Criteria of included studies; Table S4: Summary of criteria of different variables included in meta-analysis; Table S5: List of the 45 excluded studies for the sensitivity analysis; Table S6: Subgroup analysis of pooled events rates; Table S7: Subgroup analysis of pooled events rates; Table S8: Subgroup analysis of primary outcomes on lung cancer only vs. pancreatic cancer only vs. all other cancer; File S1: PRISMA 2020 Checklist.
