# Supplementary material for: Impact of Center Volume on Cardiopulmonary and Mortality Outcomes after Immune-Checkpoint Inhibitors for Cancer: A Systematic Review and Meta-Analysis
Source: Cancers (Basel). 2024 Mar 13;16(6):1136. doi: 10.3390/cancers16061136 (PMC10969050; doi:10.3390/cancers16061136)

Supplementary material

Supplementary figures legend

Supplementary Figure S1: PRISMA flowchart

Supplementary Figure S2: Bubble plots showing meta-regression of different variables on pooled events rates: A) study period on irAE grade 5, B) percentage of patients who underwent chemotherapy on cardiac related irAE, percentage of patients who underwent surgery on C) cardiac-related irAE and D) pulmonary-related irAE

Supplementary Figure S3: Funnel plots for assessment of publication bias; A for pulmonary irAEs, B for cardiac irAEs, C for grade 3,4 irAEs, D for any grade irAEs, and E for grade 5 irAEs

Supplementary Figure S4: Threshold analysis showing relation between number of annual cases and grade 3-4 (A), cardiac-related (B), and pulmonary-related (C) adverse events without 33 studies

Supplementary Figure S1: PRISMA flowchart

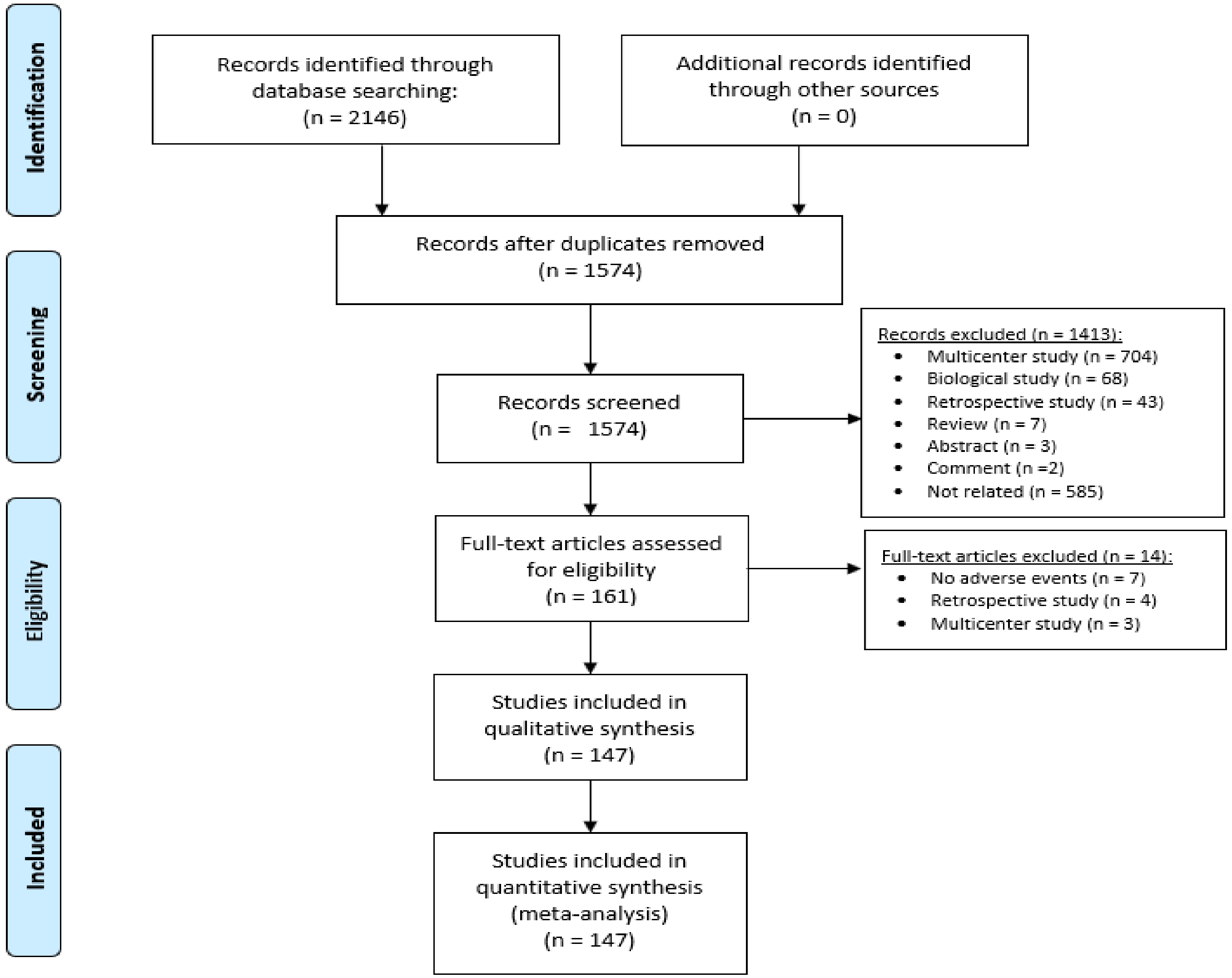

Supplementary Figure S2: Bubble plots showing meta-regression of different variables on pooled events rates: A) study period on irAEs grade 5, B) percentage of patients who underwent chemotherapy on cardiac-related irAEs, percentage of patients who underwent surgery on C) cardiac-related irAEs and D) pulmonary-related irAEs

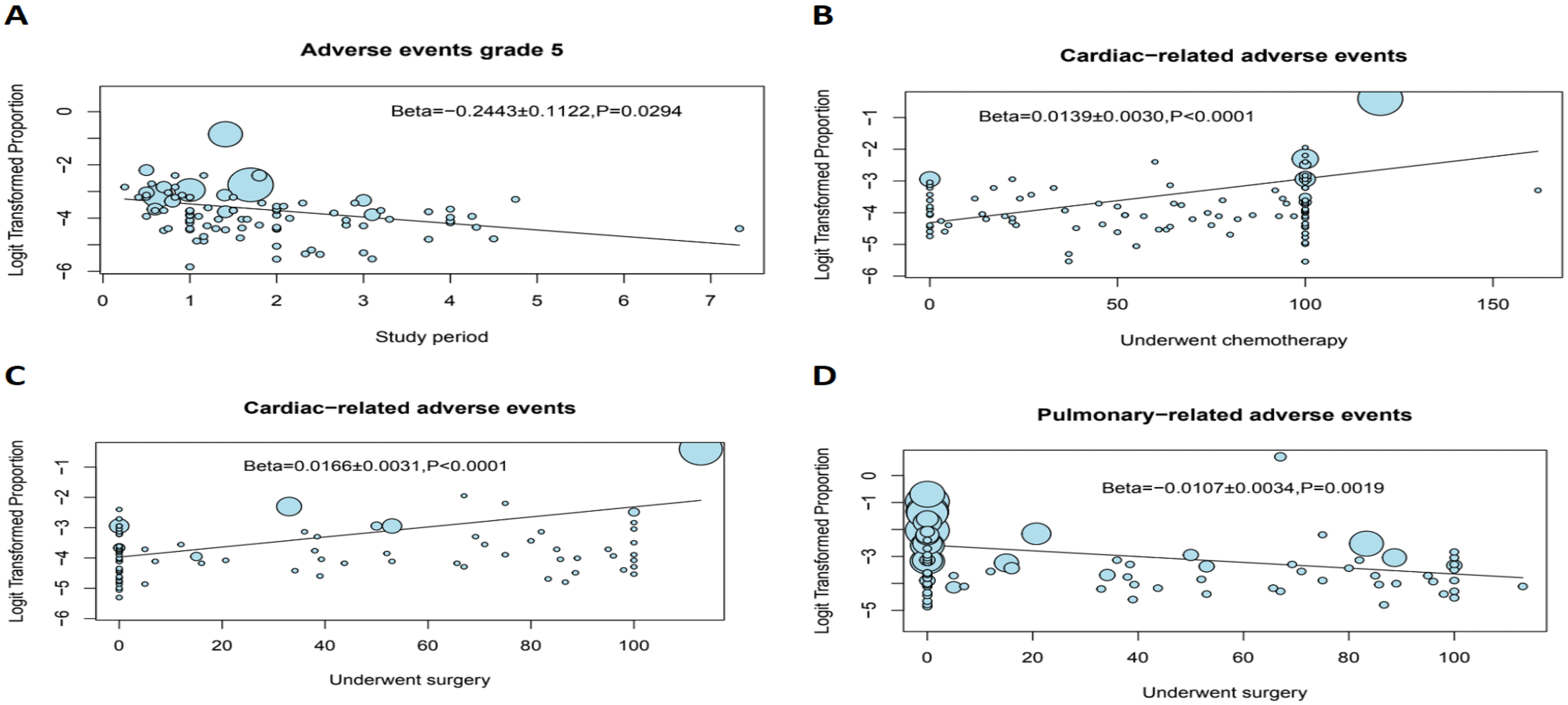

AEs: Adverse events

Supplementary Figure S3*:* Funnel plots for assessment of publication bias; A) for pulmonary irAEs, B) for cardiac irAEs, C) for grade 3,4 irAEs, D) for any grade irAEs, and E) for grade 5 irAEs

A)

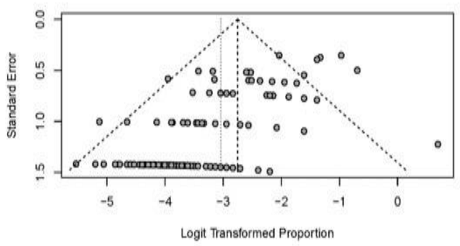

B)

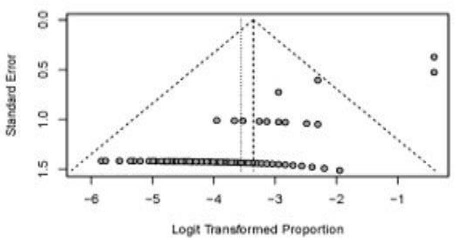

C)

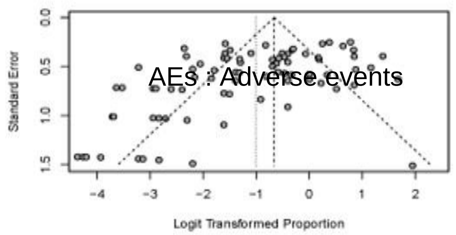

D)

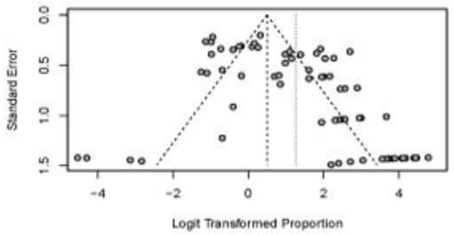

E)

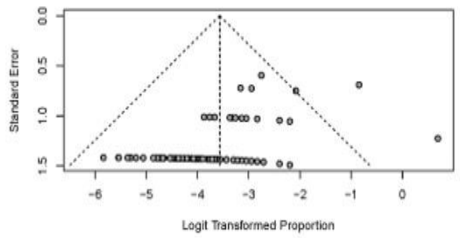

Supplementary Figure S4: Threshold analysis showing relation between number of annual cases and grade 3-4 (A), cardiac-related (B), and pulmonary-related (C) adverse events without 33 studies.

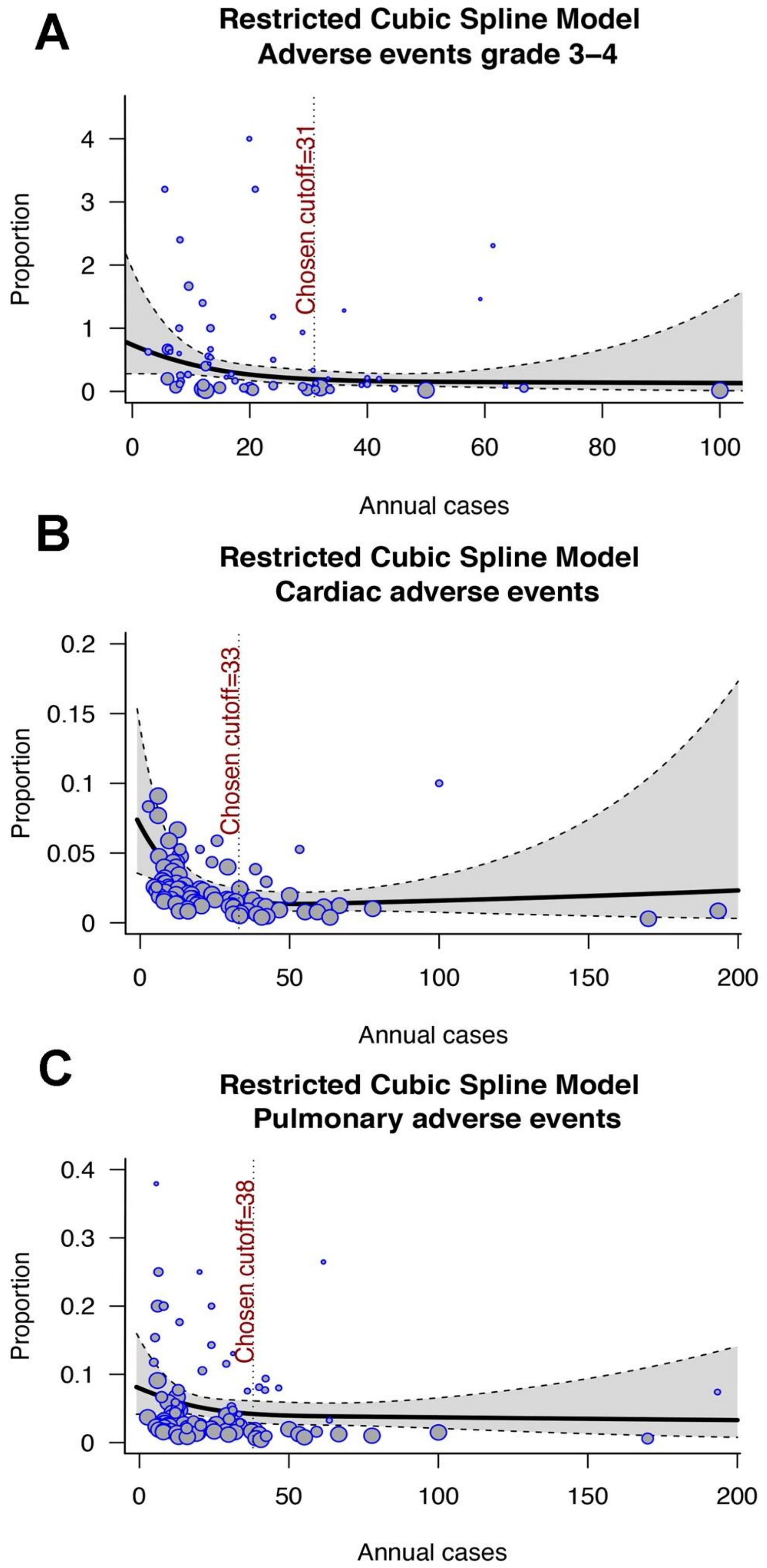

Supplement: Supplementary file 1 [file cancers-16-01136-s001.zip › Supplementary Figures.pdf]
