# Supplementary material for: Impact of Center Volume on Cardiopulmonary and Mortality Outcomes after Immune-Checkpoint Inhibitors for Cancer: A Systematic Review and Meta-Analysis
Source: Cancers (Basel). 2024 Mar 13;16(6):1136. doi: 10.3390/cancers16061136 (PMC10969050; doi:10.3390/cancers16061136)
Supplement: Supplementary file 1 [file cancers-16-01136-s001.zip › Supplementary Tables.pdf]

Supplementary material

Supplementary Tables legend

Supplementary Table S1: Search terms used in our meta-analysis

Supplementary Table S2: Quality of included studies using A) Cochrane Collaboration's tool for assessing risk of bias in randomized trials for quality assessment of randomized clinical trials (RCTs) and B) The Newcastle-Ottawa Quality Assessment Scale (NOS) for non-RCTs

Supplementary Table S3: Criteria of included studies

Supplementary Table S4: Summary of criteria of different variables included in meta-analysis

Supplementary Table S5: List of the 45 excluded studies for the sensitivity analysis

Supplementary Table S6: Subgroup analysis of pooled events rates in A) adverse events (AE) 3-4 and B) AE 5 by ITH drugs

Supplementary Table S7: Subgroup analysis of pooled events rates A) adverse events (AE) 3-4 and B) AE 5 by ITH class

Supplementary Table S8: Subgroup analysis of primary outcomes on lung cancer only vs pancreatic cancer only vs all other cancers

Supplementary Table S1: Search terms used in our meta-analysis

#	Search
**1**	"immunotherapy"[MeSH Terms] OR "immunotherapy"[All Fields] OR "immunotherapies"[All Fields] OR "immunotherapy s"[All Fields]
**2**	"anti"[All Fields] AND "pd1"[All Fields]
**3**	"anti pdl1"[All Fields]
**4**	"anti"[All Fields] AND "ctla4"[All Fields]
**5**	2 OR 3 OR 4
**6**	"atezolizumab"[Supplementary Concept] OR "atezolizumab"[All Fields]
**7**	"ipilimumab"[MeSH Terms] OR "ipilimumab"[All Fields]
**8**	"durvalumab"[Supplementary Concept] OR "durvalumab"[All Fields]
**9**	"nivolumab"[MeSH Terms] OR "nivolumab"[All Fields] OR "nivolumab s"[All Fields]
**10**	"pembrolizumab"[Supplementary Concept] OR "pembrolizumab"[All Fields]
**11**	"cemiplimab"[Supplementary Concept] OR "cemiplimab"[All Fields]
**12**	"avelumab"[Supplementary Concept] OR "avelumab"[All Fields]
**13**	6 OR 7 OR 8 OR 9 OR 10 OR 11 OR 12
**14**	1 OR 5 OR 13
**15**	"cancer s"[All Fields] OR "cancerated"[All Fields] OR "canceration"[All Fields] OR "cancerization"[All Fields] OR "cancerized"[All Fields] OR "cancerous"[All Fields] OR "neoplasms"[MeSH Terms] OR "neoplasms"[All Fields] OR "cancer"[All Fields] OR "cancers"[All Fields]
**16**	14 AND 15

Supplementary Table S2: Quality of included studies using A) Cochrane Collaboration's tool for assessing risk of bias in randomized trials for quality assessment of randomized clinical trials (RCTs) and B) The Newcastle-Ottawa Quality Assessment Scale (NOS) for non-RCTs

A

	RANDOM SEQUENCE GENERATION	ALLOCATION CONCEALMENT	BLINDING OF PARTICIPANTS	BLINDING OF OUTCOME ASSESSMENT	INCOMPLETE OUTCOME DATA	SELECTIVE REPORTING	OTHER SOURCES OF BIAS
**Altorki 2021**	+	+	+	+	+	+	+
**Amaria 2018**	+	?	-	+	+	+	+
**Arrieta 2020**	+	+	?	+	+	+	+
**Cascone 2021**	+	+	+	?	+	+	+
**Duska 2020**	+	+	+	+	+	+	+
**Ferrarotto 2020**	+	+	?	+	+	+	+
**Lee 2013**	+	?	+	+	+	+	+
**Liu 2020**	+	+	+	+	+	+	+
**Mahmood 2021**	+	+	+	+	+	+	+

Pakkala 2020	+	+	+	?	+	+	+
Sundahl 2019	+	+	+	+	+	+	+
Tobin 2018	+	+	?	+	+	+	+
Voorwerk 2019	+	+	-	+	+	+	+
Welsh 2020	+	+	+	+	+	+	+
Zhu 2022	+	+	-	-	+	+	+

+	Low Risk
?	Uncertain
-	High Risk

B

Study	Selection	Comparability	Outcome	Total
Aamdal 2021	***	*	***	*******
Ariyan 2018	***	*	***	*******
Awada 2020	****	**	***	*********
Ben-Ami 2017	***	*	***	*******
Bjoern 2016	***	*	***	*******
Boutros 2020	***	**	***	********

**Brastianos 2020**	***	*	***	*******
**Brustugun 2017**	***	*	***	*******
**Calabrò 2013**	***	*	***	*******
**Calabrò 2015**	****	*	***	********
**Calabrò 2018**	***	*	***	*******
**Chalmers 2019**	***	*	***	*******
**Chang 2021**	****	**	***	*********
**Chintakuntlawar 2019**	***	*	***	*******
**Cho 2019**	****	*	***	********
**Chu 2021**	***	*	***	*******
**Chung 2019**	***	*	***	*******
**Da Gama Duarte 2018**	***	*	***	*******
**DAngelo 2017**	****	*	***	********
**Di Giacomo 2011**	***	**	***	********
**Di Giacomo 2019**	****		***	*******
**Di Noia 2021**	***	**	***	********
**Disselhorst 2019**	****	*	***	********
**Duffy 2017**	****	*	***	********
**Edenfield 2021**	***	*	***	*******
**Eichhorn 2021**	***	*	***	*******
**Elbers 2020**	***	*	***	*******

**Feng 2020**	***	*	***	*******
**Ferrarotto 2021**	***	*	***	*******
**Feun 2019**	***	**	***	********
**Freeman-Keller 2016**	***	**	***	********
**Frumovitz 2020**	***	*	***	*******
**Gao J. 2020**	***	*	***	*******
**Gao S. 2020**	***	*	***	*******
**Giaccone 2018**	***	*	***	*******
**Goldberg 2016**	****		***	*******
**Goldberg 2020**	***	*	***	*******
**Graff 2016**	***	*	***	*******
**Graff 2020**	***	*	***	*******
**Gray 2019**	****	*	***	********
**Haag 2018**	***	*	***	*******
**Habra 2019**	***	*	***	*******
**Hamanishi 2015**	****	*	***	********
**Hasan Ali 2019**	***	*	***	*******
**Hodi 2014**	****		***	*******
**Horinouchi 2015**	****	*	***	********
**Huang 2019**	***	*	***	*******
**Kamath 2020**	***	*	***	*******
**Kanda 2016**	****		***	*******

**Kanda 2020**	****	*	***	********
**Kawazoe 2020**	***	*	***	*******
**Kelly 2020**	***	*	***	*******
**Kim 2020**	***	*	***	*******
**Kitano 2021**	****	*	***	********
**Kluger 2019**	***	*	***	*******
**Lam 2021**	***	*	***	*******
**Lin 2020**	***	*	***	*******
**Lisberg 2018**	***	*	***	*******
**Luke 2018**	***	*	***	*******
**Ma 2021**	***	*	***	*******
**Madan 2012**	***	**	**	*******
**Mahalingam 2020**	***	*	***	*******
**Maity 2018**	****		***	*******
**Maity 2021**	****	*	***	********
**Majd 2020**	***	*	***	*******
**Maker 2005**	***	*	***	*******
**Maker 2006**	***	*	***	*******
**Marcq 2021**	***	**	**	*******
**Massarelli 2019**	***	**	**	*******
**McNeel 2012**	****		***	*******
**Mego 2019**	***	*	***	*******

**Merchant 2016**	****	**	***	*******
**Miyamoto 2019**	***		**	*******
**Music 2020**	***	*	***	*******
**Naing 2020**	***	*	***	*******
**ONeill 2020**	***	*	***	*******
**Parikh 2018**	****	*	***	********
**Patel 2017**	***	*	***	*******
**Pelster 2021**	***	*	***	*******
**Perez 2021**	***	*	***	*******
**Postow 2020**	****		***	*******
**Qiao 2019**	****	*	***	********
**Qin 2017**	***	**	***	********
**Ralph 2010**	***	*	***	*******
**Ratnayake 2020**	***	*	***	*******
**Ray 2016**	***	*	***	*******
**Reilley 2017**	****		***	*******
**Reschke 2021**	****	*	**	*******
**Reuben 2006**	***	*	***	*******
**Rodriguez 2020**	****	*	***	********
**Ross 2020**	***	*	***	*******
**Royal 2010**	***	*	***	*******
**Rozeman 2020**	****		***	*******

**Sahebjam 2021**	****	*	***	********
**Sakamuri 2018**	***	*	***	*******
**Sarfaty 2021**	***	*	***	*******
**Schoenfeld 2020**	****		***	*******
**Schweizer 2020**	****	*	***	********
**Shah 2020**	****	*	***	********
**Shenderov 2021**	****	*	***	********
**Shoushtari 2018**	***	*	***	*******
**Strauss 2018**	****		***	*******
**Sundahl 2019**	***	*	***	*******
**Suresh 2019**	****		***	*******
**Takahashi 2020**	***	**	***	********
**Tang 2019**	***	**	***	********
**Thomas 2019**	***	*	***	*******
**Tree 2018**	***	**	**	*******
**Tsimberidou 2021**	***	*	***	*******
**van Dijk 2020**	****		***	*******
**Wang 2020**	****	*	***	*******
**Weber 2013**	****		***	*******
**Weber 2016**	***	*	***	*******
**Wei 2020**	****		***	*******
**Weide 2017**	***	*	***	*******

**Weiss 2017**	***	*	***	*******
**Weiss 2018**	***	*	***	*******
**Welsh 2019**	***	*	***	*******
**Welsh JW 2020**	***	*	***	*******
**Welsh J.W. 2020**	***	*	***	*******
**Wilgenhof 2016**	***	*	***	*******
**Wilky 2019**	***	*	***	*******
**Xie C. 2020**	****		***	*******
**Xie L. 2020**	***	**	***	********
**Yamamoto 2017**	***	*	***	*******
**Yang 2007**	****		***	*******
**Yang 2018**	****	*	**	*******
**Yarchoan 2020**	***	*	***	*
**Yuan 2021**	***	**	***	********
**Zamarin 2020**	***	*	***	*******
**Zhang 2021**	***	*	***	*******
**Zimmer 2019**	***	*	***	*******

Supplementary Table S3: Criteria of included studies,

Author/year	Stud [illegible]	Pati [illegible]	Country	Cancer type	Mea [illegible]	Male [illegible]	Pathology (commonest [illegible]	Unde [illegible]	Re [illegible]	Re [illegible]	ITH drug	Target	Mean follow- [illegible]	Overall [illegible]	Progression- [illegible]	AE
**Lam 2021 (1)**	P	40	China	Lung cancer	61.5	20	Lung cancer	N/A	13	9	atezolizuma [illegible]	PD-L1	17.8 months	Median [illegible]	9.4 (95% [illegible]	2
**Edenfield 2021 (2)**	P	49	USA	Rare solid cancers	62	22	Cholangiocarcinoma 12 (34)	N/A	N/A	0	N/A	N/A	18 months	N/A	N/A	N/A
**Ferrarotto 2021 (3)**	P	20	USA	Skin Cancer	68	17	SCC	17	13	13	pembrolizu mab	PD-1	44.1 months	8.31 months	2.04 months	N/A
**Zhu 2022 (4)**	RCT	170	China	Pancreatic cancer	N/A	105	ADC	N/A	N/A	N/A	pembrolizu [illegible]	PD-1	23.3 months	24·9 months [illegible]	18·3 months [illegible]	N/A
**Reschke 2021 (5)**	P	17	Germany	Skin Cancer	70	9	Melanoma	N/A	2	2			Follow-up of patients was after at least 6 months (maximum 18 [illegible]	N/A	N/A	N/A
**Chang 2021 (6)**	P	25	Taiwan	solid cancers	54	21	Not specified	N/A	17	25	pembrolizu mab	PD-1	Not reported	8.5 months (95% CI, 6.6~ not [illegible]	1.9 months (95% CI, 1.9 ~ 6) by RECIST [illegible]	N/A
**Zhang 2021 (7)**	RCT	20	China	Esophageal cancer	64	12	SCC	N/A	20	N/A	camrelizum ab	PD-1	31.0 months	16.7 months	11.7 months	N/A
**Tsimberidou 2021 (8)**	P	12	USA	Germ cell tumors	35	83.3	Testicular germ cell tumor, 7(58.3%)	0	0	12	pembrolizu mab	PD-1	N/A	10.6 (95% CI, 4.6–27.1)	2.4 (95% [CI], 1.5–4.5)	6
**Marcq 2021 (9)**	P	8	Canada	Bladder Cancer	68	75	Bladder Cancer	8	8	8	atezolizuma b	PD-L1	N/A	N/A	N/A	5
**Altorki 2021 (10)**	RCT	60	USA	Lung cancer	70.5	51.6	**ADC**: 34(56.6%)	52	30	N/A	durvalumab	PD-L1	16·9 months	N/A	N/A	11

**Di Noia 2021 (11)**	RCT	42	Italy	Lung cancer	70.5	71	Non-SCC: 36 (86%)	0	24	0	pembrolizumab	PD-1	18.5 mo	Not reached in group 1&2<br>Group 3: 7.2 (95% CI 5.6 [illegible]	Median PFS for all groups : 8 (95% CI: 4.4–17.4)	1
**Aamdal 2021 (12)**	P	12	Norway	Melanoma	57	58	Melanoma	0	0	4	ipilimumab	CTLA-4	61.0 months	not reached	6.7	5
**Shenderov 2021 (13)**	P	30	USA	Prostate Cancer	67	100	ADC	0	0	30	N/A	N/A	9.9 (range, 2.3 –17.3)	9.5 (8.1, 11.4)	3.0 (2.7, 5.5)	12
**Sahebjam 2021 (14)**	P	32	USA	High grade Gliomas	52.5	65.6	Glioblastoma: 90.6%	14	32	32	pembrolizumab	PD-1	Group A: 3.7 months<br>Group B: 3.1 months	Group A: 13.45 months<br>Group B: 9.3 months	Group A: 7.92 months<br>Group B: 6.54 months	12
**Chu 2021 (15)**	P	22	China	Lung cancer	64.5	95.5	SCC 54.5%	0	0	0	sintilimab	PD-1	15.8 months	N/A	15 months	12
**Ma 2021 (16)**	P	39	China	Melanoma or nasopharyngeal carcinoma	48	72	Nasopharyngeal carcinoma: 76%	23	19	25	ipilimumab	CTLA-4	Group A: 4.9<br>Group B: 4.8	Group A: Not reached<br>Group B: 76.6%	Group A: 10.1<br>Group B: 14.3%	0

**Cascone 2021 (17)**	RCT	44	USA	Lung cancer	65.6	64	ADC: 59%	39	4	17	Nivolumab & Ipilimumab	PD-1 & CTLA-4	22.2 months	Group A: 22 patients alive at end of follow up Group B: 21 Patients alive	Group A: 19 Patients progression free Group B: 16 Patients Progression free	5
**Eichhorn 2021 (18)**	P	15	Germany	Lung cancer	59.8	46.6	ADC: 86.6%	12	0	0	pembrolizu mab	PD-1	N/A	N/A	N/A	3
**Pelster 2021 (19)**	P	35	United States.	Skin Cancer	62	12	Melanoma	N/A	N/A	1	N/A	N/A	13.0 months	19.1 months.	5.5 months	0
**Sarfaty 2021 (20)**	P	13	United States.	carcinoma of the urinary tract	57	10	SCC<br>ADC	5	N/A	21	N/A	N/A	7.38 months	6.97 months	1.8 months	N/A
**Yuan 2021 (21)**	P	16	United States.	Breast cancer	64	0	Triple -ve Breast cancer	16	15	N/A	pembrolizu mab	PD-1	24.9 months	2.6 months	25.5 months	N/A
**Perez 2021 (22)**	P	21	USA	Lung cancer	66	62	SCLC	0	21	21	N/A	N/A	N/A	11.7 months	4.5 months	16
**Maity 2021** [illegible]	P	22	USA	Melanoma	N/A	77.2	Melanoma	0	22	11	ipilimumab	CTLA-4	89.2 months [illegible]	10.7 months [illegible]	3.6 months [illegible]	3
**Kitano 2021 (24)**	P	13	Japan	Lung: 15.4%<br>Bladder: 7.7%<br>Breast: 7.7)<br>Urethra: 1 (7.7%)<br>Uterus: 1 (7.7%)<br>Ovary: 1 (7.7%)<br>prostate: 1 (7.7%)<br>[illegible]	62	38.4	N/A	9	7	12	cemiplimab	PD-1	8.11 (range 2.0–26.1).	N/A	N/A	4
**Mahmood 2021 (25)**	RCT	20	USA	Adenoid Cystic Carcinoma	64.5	20	Adenoid Cystic Carcinoma	19	17	9	pembrolizu mab	PD-1	19.8 months	Not reached	Arm A: 4.5 months (95%[CI], 2.4-20.6)<br>[illegible]	1
**Pakkala 2020 (26)**	RCT	18	USA	Lung Cancer	70.00	61.1	SCLC	0	0	18	Durvalumab + Tremelimum	PD-L1 & CTLA-4	N/A	Median OS:3.9	Median PFS: 3.16.00	N/A

**Majd 2020 (27)**	P	4	USA	Pituitary carcinoma	27.5	50	corticotroph adenoma: 3 Lactotroph adenoma : 1	3	3	4	pembrolizu mab	PD-1	N/A	80	8	0
**van Dijk 2020 (28)**	P	24	Netherland s	Urinary Bladder Cancer	65	75	Urothelial Carcinoma	24	0	0	N/A	N/A	8.3 months	Obtained from Kaplan Meier curve	Obtained from Kaplan Meier curve	13
**Gao 2020 (29)**	P	28	USA	Urinary Bladder Cancer	71	71	Pure urothelial carcinoma 21 (75%)	24	0	0	N/A	N/A	19.2	88.8%	82.8%	6
**Welsh 2020 (30)**	P	40	USA	Lung cancer	64	40	SCLC: 36 (90%)	0	40	40	pembrolizu mab	PD-1	23.1 months	39.5 months	19.7 months	N/A
**Duska 2020 (31)**	RCT	52	USA	Cancer Cervix	49	0	squamous cell carcinoma	0	52	52	pembrolizu mab	PD-1	4.8 months	N/A	N/A	34
**Schweizer 2020 (32)**	P	104	Germany	HNSCC 44 (42%) Lung Cancer 46 (44%)	N/A	73	Squamous cell Carcinoma 64 (65%)	0	50	6	N/A	N/A	8.3 months.	22.8 months	7.8 months	4
**Takahashi 2020 (33)**	P	57	Japan	Melanoma	58	49.1	Melanoma	0	0	0	N/A	N/A	6.75 months	14 months	3.3 months	32
**Awada 2020 (34)**	P	54	Belgium	Glioblastoma	55	63	Glioblastoma	45	45	43	avelumab	PD-L1	24.9	26.6	Cohort 1: 12.0	N/A
**Welsh 2020 (35)**	RCT	100	USA	Lung cancer	65	64	ADC: 77(77%)	0	18	37	pembrolizu mab	PD-1	20.4 (1.4–30.2)	N/A	SBRT Group: 20.8 RT Group: 6.8 Pembrolizuma b Alone: 6.8	17

**Wang 2020 (36)**	P	41	China	Lung Cancer	59	70.7	ADC: 29 (70.7%)<br>SCC: 10 (24.4%)	14	20	41	toripalimab	PD-1	14.9 (3.2-22.5) months	13.8 months (95% CI, 10.0	2.8 (95% CI, 2.7 to 4.6)	1
**Schoenfeld 2020 (37)**	P	29	USA	HNSCC	65.2	62.1	SCC	29	0	0	N/A	N/A	14.2 months	89%	85%	N/A
**ONeill 2020 (38)**	P	10	USA	Pancreatic Cancer	62	40	Adenocarcinoma	10	N/A	10	nivolumab	PD-1	12 Months	18.0 months	6.8 months	7
**Frumovitz 2020 (39)**	P	7	USA	Gynecologic small cell neuroendocrine carcinoma	41	0	Small cell Neuroendocrine Carcinoma	0	N/A	N/A	pembrolizu mab	PD-1	N/A	N/A	2.1 months (Median)	2
**Ratnayake 2020 (40)**	P	24	Austrialia	Melanoma	66.8	79	N/A	0	24	0	N/A	N/A	28 months	16.9 months	2.2 months	N/A
**Boutros 2020 (41)**	P	19	France	Melanoma	58	53	N/A	N/A	19	N/A	ipilimumab	CTLA-4	5.8 years (Q1=4.5; Q3=6.8)	0.9 years (0.5–2)	0.4 (0.2–1.4)	N/A
**Kawazoe 2020 (42)**	P	29	Japan	Gastric and esophageal	70	90	Intestinal: 15 (52%)	6	0	15	pembrolizu mab	PD-1	12.6 (10.5-14.3)	not reached	7.1 (5.4-13.7)	14
**Wei 2020 (43)**	P	25	China	Solid tumors	52	80	N/A	N/A	N/A	25	toripalimab	PD-1	5mo (1.5-19.8)	N/A	N/A	0
**Brastianos 2020 (44)**	P	20	USA	Solid tumors	51.5	0	N/A	10	18	20	pembrolizu mab	PD-1	N/A	3.6 months	N/A	8
**Kim 2020 (45)**	P	9	USA	lung cancer or high grade neuroendocrine	61	44	SCLC: 6 (67%)	0	3	9	nivolumab	PD-1	6.8 mos	N/A	N/A	5
**Graff 2020 (46)**	P	28	USA	Prostate	72	100	Not specified	11	8	4	pembrolizu mab	PD-1	37 months	21.9 months	N/A	N/A
**Ferrarotto 2020 (47)**	RCT	28	USA	Oropharyngeal	N/A	97	N/A	0	9	0	N/A	N/A	15.8 months	100% still alive	N/A	4
**Postow 2020 (48)**	P	20	USA	Melanoma	60	60	N/A	0	10	N/A	N/A	N/A	19.4 months	1 year OS 56.3%	N/A	7

**Zamarin 2020 (49)**	P	27	USA	Ovarian	64	0	High grade serous: 23 (85.2%)	0	0	27	durvalumab	PD-L1	29 months	21 months	2.8 months	N/A
**Feng 2020 (50)**	P	32	China	Hepatobiliary	60	56	N/A	21	10	7	nivolumab	PD-1	12.8 months	8.5 months	6.1 months	N/A
**Kanda 2020 (51)**	P	24	Japan	Lung Cancer	63	70.8	ADC: 79.2%	N/A	N/A	N/A	nivolumab	PD-1	N/A	N/A	N/A	2
**Arrieta 2020 (52)**	RCT	78	Mexico	Lung Cancer	50.1	41	ADC: 88%	0	0	43	pembrolizu mab	PD-1	8.9 months	14.8	9.5 months	2
**Rozeman 2020 (53)**	P	38	Netherland s	Melanoma	62	59	N/A	N/A	N/A	N/A	ipilimumab	CTLA-4	N/A	12.4	3 mos	16
**Xie 2020 (54)**	P	59	USA	pancreatic	61	63	N/A	0	0	59	durvalumab	PD-L1	N/A	A1: 3.3 mos (1.2-6.6) A2:9 (.5-18.4) [illegible]	A1: 1.7mos (0.8-2.0) A2: 2.5 (0.1- [illegible]	18
**Music 2020 (55)**	P	78	Canada	solid tumors	61	45	High Grade serous ovarian carcinoma	N/A	N/A	N/A	pembrolizu mab	PD-1	N/A	23.8 mo	N/A	7
**Liu 2020 (56)**	RCT	40	China	Breast	45.5	0	Triple negative	N/A	N/A	30	camrelizum ab	PD-1	N/A	8.1 mos (4.0-not reached)	3.7 mos (2.0-6.4)	10
**Xie 2020 (57)**	P	43	China	Osteosarcoma	19	73	N/A	0	0	43	camrelizum [illegible]	PD-1	11.3 (7.1- [illegible]	11.3 mos [illegible]	6.2 mos(4- [illegible]	30
**Goldberg 2020 (58)**	P	42	USA	Lung Cancer	60	33	ADC: 86%	N/A	N/A	27	pembrolizu mab	PD-1	8.3 mos (4.5-26.2)	1yr OS 40% (30-64%)	1.9 (1.8-3.7)	7
**Gao 2020 (59)**	P	40	China	Lung Cancer	59.8	82.5	SCC: 82.5%	0	0	0	sintilimab	PD-1	3.2 mos(1.2-12.1)	N/A	N/A	4.0 0
**Kamath 2020 (60)**	P	21	USA	Pancreatic Cancer	66	38	ADC: 86%	8	4	14	ipilimumab	CTLA-4	N/A	6.9 mos (2.6-9.6)	2.78mos (1.6-4.8)	16. 00
**Naing 2020 (61)**	P	127	USA	Multiple solid tumor	56	54	N/A	N/A	N/A	127	pembrolizu mab	PD-1	N/A	N/A	N/A	11. 00
**Kelly 2020 (62)**	P	20	USA	Sarcoma	63.5	40	N/A	0	0	19	pembrolizu mab	PD-1	56 weeks	74.7wks (49-n/a)	17.1 wks (12.6-n/a)	4
**Ross 2020 (63)**	P	12	USA	Prostate	65.5	100	N/A	0	0	0	pembrolizu mab	PD-1	31.3 mos (18.1-39.3)	N/A	17.5mos (not specified)	0

**Rodriguez 2020 (64)**	P	50	USA	HEENT	60.5	78	N/A	0	0	25	pembrolizumab	PD-1	12.6 mo	12.6mos (8.1-not reached)	4.5 mos (4.1-8.4)	N/A
**Shah 2020 (65)**	P	30	USA	Breast	51	0	HR+, HER-, or triple negative	0	0	23	pembrolizumab	PD-1	N/A	15.4 mos (8.2-20.3	4.0 mos (2-6.4)	N/A
**Yarchoan 2020 (66)**	P	12	USA	Colorectal	58	35	N/A	0	11	12	pembrolizumab	PD-1	N/A	213 days (179-441)	82 days (48-97)	2
**Lin 2020 (67)**	P	40	USA	Lung Cancer	67	68	ADC: 55%	0	0	0	atezolizumab	PD-L1	part I: 22.5mos (19.0-29.1) Part II: 15.3 mos (10.9-	part I: 22.8 Part II: not reached	Part I: 12.5mo Part II: 13.2mos	32.00
**Welsh 2020 (68)**	P	38	USA	Lung Cancer	62	61	Small cell neuroendocrine: 91%	0	0	38	pembrolizumab	PD-1	7.3mos (1-13)	8.4 (6.7-10.1)	6.1mos (4.1-8.1)	2.00
**Mahalingam 2020 (69)**	P	11	USA	Pancreatic	64	27	N/A	4	11	7	pembrolizumab	PD-1	N/A	3.1 mos (0-8.7)	2.0 mos (0-6.8)	2.00
**Elbers 2020 (70)**	P	10	Netherlands	HNSCC	69	8	SCC of the oral cavity, oropharynx, hypopharynx or larynx	N/A	9	N/A	avelumab	PD-L1	12 (8–26)	N/A	N/A	4
**Di Giacomo 2019 (71)**	P	19	Italy	melanoma	58	16	N/A	N/A	N/A	N/A	ipilimumab	CTLA-4	26.3 months	26.2 months (95% CI, 3.5–48.9);	5.6 months (95% CI, 4.5–6.6)	16
**Welsh 2019 (72)**	P	106	USA	Non Melanoma metastatic cancer	60	50	ADC 57, SCC 13, Neuroendocrine 9, Sarcoma 7, Adrenocortical 5, Other 15	N/A	106	N/A	ipilimumab	CTLA-4	10.5 months	median overall survival time was not [illegible]	2.9 months	N/A
**Gray 2019 (73)**	P	33	USA	Lung Cancer	68	22	ADC 25, Adenosquamos Carcinoma 1, Squamos cell carcinoma 7	N/A	N/A	27	pembrolizumab	PD-1	18.2 months	Immunotherapy Refractoty: 6.8 (95% CI,	Immuntherapy Refractory: 2.8 (95% CI, 1.8—not	7.00
**Qiao 2019 (74)**	P	11	China	pancreatic, gastric, colorectal, cervical, endometrial cancer and peritoneal cancer	N/A	45	pancreatic: 1 gastric: 1 colorectal: 4 cervical:2 endometrial: 2 Peritoneal: 1	6	3	7	pembrolizumab	PD-1	N/A	N/A	N/A	0

**Chintakuntlawar 2019 (75)**	P	3	USA	Anaplastic thyroid cancer	56	33	Anaplastic thyroid cancer: Sarcomatoid: 1 (33%) Epithelial: 1 (33%) Unspecified: 1 (33%)	2	3	3	pembrolizumab	PD-1	N/a	2.76 months	N/A	3
**Feun 2019 (76)**	P	29	USA	Hepatocellular Carcinoma	67	25	N/A	N/A	1	N/A	pembrolizumab	PD-1	17 months	13 months	4.5 months	3
**Habra 2019 (77)**	P	16	USA	adrenocortical carcinoma	48	8	ACC	N/A	N/A	N/A	pembrolizumab	PD-1	N/A	N/A	N/A	1
**Cho 2019 (78)**	P	33	Korea	Thymoma=7, Thymic carcinoma=26	57	21	73.1% SCC	11	12	33	pembrolizumab	PD-1	14.9 months	N/A	6.1 months (5.3-6.9)	0
**Chalmers 2019 (79)**	P	14	USA	Lung Cancer (NSCLCs)	N/A	N/A	N/A	N/A	N/A	N/A	ipilimumab	CTLA-4	n/a	> 42.3 months	24.1 month	5
**Mego 2019 (80)**	P	8	Slovakia	Germ cell tumor	29	100	1 embryonal carcinoma, 1 choriocarcinoma, 1 yolk sac tumor, 1 immature	0	0	8	avelumab	PD-L1	2.6 (0.3 to 14.4)	2.7 months (95% CI 1.0–3.3)	0.9 months (95% CI 0.5–1.9)	0
**Thomas 2019 (81)**	P	20	USA	Lung Cancermall Cell Lung Cancer	64	45	N/A	1	9	20	durvalumab	PD-L1	11.1 months	4.1 months	1.8 months	N/A
**Zimmer 2019 (82)**	P	9	USA	7 ovarian/1 endometrial/1 triple negative breast cancer	59	0	OvCa (HGSOC/Clear cell/Mixed Mullerian/ Mixed Serous and Endometrioid) 6 (2/2/1/1) Platinum sensitivity in OvCa (sensitive/resistant) 2/5 Primary Peritoneal cancer (platinum-resistant) 1 Endometrial carcinoma (MSI low) 1 Triple Negative Breast Cancer 1	0	0	2	durvalumab	PD-L1	8 months	N/A	N/A	N/A

**Suresh 2019 (83)**	P	18	USA	Melanoma, BCC, Lung Cancer	71	72	Melanoma: 2(11.1%) NSCLC: 15(83.3%) BCC: 1(5.5%)	0	8	14	N/A	N/A	N/A	N/A	N/A	6
**Sundahl 2019 (84)**	P	20	Belgium	Melanoma	60.5	65	N/A	N/A	20	N/A	nivolumab	PD-1	13.1	N/A	6.5 months	N/A
**Voorwerk 2019 (85)**	RCT	70	Netherlands	Breast	51	0	N/A	N/A	N/A	70	nivolumab	PD-1	19.9 months	N/A	1.9 months	2
**Wilky 2019 (86)**	P	33	USA	Advanced or metastatic sarcomas,	44	55	N/A	N/A	N/A	5	pembrolizumab	PD-1	14.7 months	18.7	6.9	N/A
**Sundahl 2019 (87)**	RCT	18	Belgium	Urothelial carcinoma	64.5	88.8	Urothelial Carcinoma	0	18	0	pembrolizumab	PD-1	2.8 Months	Arm A: 4.5 Arm B: 12.1	Arm A: 3.3 Arm B: 3.5	1
**Disselhorst 2019 (88)**	P	35	Netherlands	pleural mesothelioma	65	77	N/A	N/A	N/A	35	N/A	N/A	14.3	N/A	6.2	N/A
**Huang 2019 (89)**	P	30	China	Esophageal squamous cell [illegible]	60.5	76.7	N/A	16	3	29	camrelizumab	PD-1	28.7	N/A	8 weeks	N/A
**Chung 2019 (90)**	P	11	USA	1 hepatocellular carcinoma, 1 head and neck squamous cell carcinoma, 2 pancreatic ductal adenocarcinoma and 7 triple negative	57.7	36	N/A	N/A	N/A	N/A	pembrolizumab	PD-1	N/A	N/A	N/A	N/A
**Miyamoto 2019 (91)**	P	6	Japan	Lung Cancer	60.2	66.7	N/A	N/A	6	N/A	nivolumab	PD-1	N/A	N/A	3.36	1
**Tang 2019 (92)**	P	30	China	Melanoma or Urologic	51	58.3	N/A	34	7	36	toripalimab	PD-1	5.8 months	12.2 months	2.8 months	21
**Hasan Ali 2019 (93)**	P	102	Switzerland	Lung Cancer: 66 (65%) Melanoma: [illegible]	68	51	N/A	N/A	N/A	N/A	N/A	N/A	N/A	N/A	N/A	N/A

**Kluger 2019 (94)**	P	23	USA	melanoma	65	65	N/A	12	12	N/A	pembrolizumab	PD-1	34 months	17 months	2 months	N/A
**Massarelli 2019 (95)**	P	24	USA	22 (92) oropharyngeal cancer, Cervix1 (4) Anus1 (4)	60	83	N/A	N/A	N/A	N/A	nivolumab	PD-1	12.2	17.5 months	2.7 months	N/A
**Parikh 2018 (96)**	P	12	USA	urothelial carcinoma, recurrent or advanced disease	66	8	urothelial carcinoma	N/A	N/A	2	pembrolizumab	PD-1	Arm A 5.7 months, Arm B 3.7 months	N/A	4.8 months	7.00
**Maity 2018 (97)**	P	24	USA	Melanoma and others (pancreas and breast)	59.7	10.42	N/A	18	15	N/A	pembrolizumab	PD-1	N/A	N/A	N/A	8
**Amaria 2018 (98)**	RCT	53	USA	Melanoma	52	35.8	Superficial spreading (42%)	8	1		Nivolumab: + Ipilimumab	PD-1 & CTLA-4	15.0 months	100% at 24.4 months with I+N versus 76% (31–94%) at 22.6 mo with N, p=0.18	82% at 17.2 months (mo) with I+N versus 58% (27–80%) at 22.6 mo with N	9
**Tobin 2018 (99)**	RCT	10	USA	Melanoma	52.1	40	N/A	0	0	0	ipilimumab	CTLA-4	group 1 318 days group 2 365 days	N/A	N/A	5
**Tree 2018 (100)**	P	5	UK	Urinary Bladder			N/A	N/A	N/A	5	pembrolizumab	PD-1	N/A	N/A	N/A	2.00
**Lisberg 2018 (101)**	P	11	USA	Lung Cancer	58.7	36.3	Squamous 1 Non squamos 10	0	0	0	pembrolizumab	PD-1	233 days	N/A	N/A	1

**Calabrò 2018 (102)**	P	40	Italy	pleural or peritoneal mesothelioma	64	73	epithelioid: 32 (80%)<br>Sarcomatoid: 2 (5%)<br>Biphasic: 5 (13%)	N/A	N/A	N/A	N/A	N/A	N/A	16.6 months	5.7 moths	7
**Luke 2018 (103)**	P	73	USA	Ovarian 9, Lung Cancer 7, breast 6, cholangiocarcinoma 6, endometrial 6, colorectal 5, head and neck 4, others	62	39.7	N/A	0	69	73	pembrolizu mab	PD-1	5.5 months	9.6 months	PFS was 3.1 months	50
**Strauss 2018 (104)**	P	19	Germany	adenoid cystic carcinoma 2, anal 2, Appendiceal 1, bronchopulmonary carcinoid 1, cervix 4, chordoma 1, colorectal 2	56	52.6	N/A	0	0	19	m7824	PD-L1	N/A	N/A	N/A	4
**Da Gama Duarte 2018 (105)**	P	5	Australia	Melanoma	59	60	Melanoma	0	0	3	ipilimumab	CTLA-4	study terminated due to	study terminated due to	study terminated due to	2
**Giaccone 2018 (106)**	P	40	USA	Thymic carcinoma	57	70	SCC 48% ,<br>Poorly differentiated 32%.<br>Neuroendocrine 15%<br>Not specified 5%	21	23	40	pembrolizu mab	PD-1	20 months (IQR 14–26)	24·9 months (15·5–not reached)	4·2(95% CI 2·9–10·3)	N/A
**Yang 2018 (107)**	P	13	USA	Lung Cancer	61.4	38	ADC:62%,<br>SCC:38%	13	0	13	ipilimumab	CTLA-4	N/A	29.2 (95% CI: 22.1 to not reached)	N/A	5
**Sakamuri 2018 (108)**	P	36	USA	Melanoma, Thyroid, Adrenocortical cancer, Adenoid cystic cancer, Leiomyosarcoma, Hodgkin Lymphoma, Renal cancer, Others	56	33	Hodgkin Lymphoma 7 (19%)	N/A	N/A	N/A	ipilimumab	CTLA-4	N/A	N/A	N/A	N/A
**Ariyan 2018 (109)**	P	26	USA	Melanoma	N/A	62	Melanoma	N/A	N/A	26	ipilimumab	CTLA-4	36 Months	N/A	N/A	10

**Haag 2018 (110)**	P	25	Germany	Melanoma	64	80	Melanoma	24	5	9	ipilimumab	CTLA-4	4.56(2.2---14.2)	22.7 (95% CI 9.5-Not [illegible]	2.9 (95% CI 2.5e8.1)	9
**Weiss 2018 (111)**	P	17	USA	Pancreatic Cancer	55	35.3	ADC	2	1	4	pembrolizumab	PD-1	N/A	15 (95%CI, 6.8----22.6)	9.1(95%CI, 4.9---15.3)	12
**Shoushtari 2018 (112)**	P	64	USA	Melanoma	56	50	Melanoma	3	3	N/A	N/A	N/A	14 months	N/A	N/A	38
**Patel 2017 (113)**	P	64	USA	Melanoma	62	70	Melanoma	0	0	N/A	ipilimumab	CTLA-4	20 months (2–60)	Not reached	5	N/A
**Ben-Ami 2017 (114)**	P	12	Israel	Uterus	54.5	N/A	Leiomyosarcoma	N/A	N/A	N/A	nivolumab	PD-1	Not reported	Because of the small [illegible]	1.8 months (95% [illegible]	N/A
**Qin 2017 (115)**	P	24	USA	Lung	64	24	NSCLC	N/A	18	24	pembrolizumab	PD-1	9.2 months	Not reported	Not reported	N/A
**Weiss 2017 (116)**	P	49	USA	Breast, pancreatic, NSCLC, Sarcoma, SCLC, ovarian cancer, and other	55	13	Breast cancer	19	16	2	pembrolizumab	PD-1	N/A	10.46 months	N/A	N/A
**DAngelo 2017 (117)**	P	28	USA	Gastric	56	17	GIST & other sarcomas	N/A	N/A	N/A	ipilimumab	CTLA-4	17.5 months	13.5 months	2.8 months	N/A
**Reilley 2017 (118)**	P	35	USA	Gastric, Renal, Skin & Lung	57.5	23	Melanoma	N/A	N/A	N/A	ipilimumab	CTLA-4	Not reported	Not reported	N/A	N/A
**Weide 2017 (119)**	P	15	Germany	Skin	54	9	Melanoma	N/A	N/A	4	ipilimumab	CTLA-4	231 days	12 months	N/A	N/A
**Yamamoto 2017 (120)**	P	17	Japan.	Colorectal. Lung	N/A	10	ADC	12	5	16	nivolumab	PD-1	24 months	Not reported	N/A	N/A

**Brustugun 2017 (121)**	P	58	Norway	Lung cancer	64.6	28	ADC 32 (55.2)	N/A	N/A	N/A	nivolumab	PD-1	14.3 months	11.7 months	N/A	N/A
**Duffy 2017 (122)**	P	32	USA	Liver cancer	61	28	Hepatocellular carcinoma	5	5		tremelimumab	CTLA-4	18.8 months	12.3 months	Six and 12-month tumor [illegible]	N/A
**Kanda 2016 (123)**	P	24	Japan	Lung cancer	63	17	NSCLC	N/A	N/A	N/A	nivolumab	PD-1	6.23 months (range, 2.04– [illegible]	Not reported	6.28 months in arm A, 9.63 [illegible]	N/A
**Ray 2016 (124)**	P	12	United [illegible]	Skin cancer	63.5	6	Melanoma	N/A	N/A	N/A	ipilimumab	CTLA-4	N/A	>31 months	N/A	N/A
**Graff 2016 (125)**	P	10	USA	prostate cancer	72	10	ADC	N/A	N/A	N/A	pembrolizumab	PD-1	7.5 months	N/A	Three patients remain free of [illegible]	N/A
**Bjoern 2016 (126)**	P	10	Denmark	Skin cancer	51.1	8	Melanoma	N/A	N/A	N/A	ipilimumab	CTLA-4	N/A	N/A	N/A	N/A
**Goldberg 2016 (127)**	P	36	USA	skin and lung cancer	62	18	Melanoma and NSCLC	24	9	N/A	pembrolizumab	PD-1	Melanoma>> 11·6 months. [illegible]	Melanoma was not [illegible]	N/A	N/A
**Wilgenhof 2016 (128)**	P	39	Netherland	skin cancer	46	23	Melanoma	N/A	22	18	ipilimumab	CTLA-4	36 months	14.75 months	6.75 months	N/A
**Weber 2016 (129)**	P	126	USA	Skin cancer	60	60	Melanoma	N/A	N/A	47	nivolumab	PD-1	16 months	20.6 months	5.3 months	N/A
**Merchant 2016 (130)**	P	33	USA	Metastatic skin cancer from many solid tumors.	N/A	14	Melanoma<br>Sarcoma<br>Renal/bladder carcinoma	N/A	17	23	ipilimumab	CTLA-4	Not reported	irAE n= 14 >> 100 % 3months,	N/A	N/A
**Freeman-Keller 2016 (131)**	P	159	USA	Skin	N/A	N/A	Melanoma	N/A	N/A	N/A	nivolumab	PD-1	35months	16.8 months	N/A	N/A
**Hamanishi 2015 (132)**	P	20	Japan	Ovarian cancer	61.1	0	Serous>	N/A	N/A	20	nivolumab	PD-1	11.0 months	20 months.	3.5 months	N/A
**Horinouchi 2015 (133)**	P	15	Japan.	Lung cancer	61	12	NSCLC	N/A	N/A	N/A	ipilimumab	CTLA-4	N/A	9 months	N/A	N/A

**Calabrò 2015 (134)**	P	29	Italy.	Lung cancer	65	20	Mesothelioma	N/A	N/A	15	tremelimumab	CTLA-4	21·3 months	11.3 months	6.2 months	2
**Hodi 2014 (135)**	P	46	USA	Skin cancer	58	28	Melanoma	N/A	20	29	ipilimumab	CTLA-4	17.3 months	25.1 months.	N/A	N/A
**Weber 2013 (136)**	P	90	USA	Skin cancer	N/A	58	Melanoma	N/A	N/A	N/A	nivolumab	PD-1	8.1 months	N/A	N/A	N/A
**Calabrò 2013 (137)**	P	29	Italy.	Skin cancer	64	21	Mesothelioma.	N/A	N/A	25	tremelimumab	CTLA-4	27 months	10.7 months	6.2 months	N/A
**Le 2013 (138)**	RCT	30	USA	Pancreatic cancer	62	20	ADC.	N/A	N/A	28	ipilimumab	CTLA-4	25 months	4.3 months	Not reported	N/A
**McNeel 2012 (139)**	P	11	USA	Prostatic cancer	63	11	ADC.	9	6	N/A	tremelimumab	CTLA-4	15 months	N/A	N/A	N/A
**Madan 2012 (140)**	P	30	USA	Prostatic cancer	69	30	ADC.	N/A	N/A	6	ipilimumab	CTLA-4	Not reported.	34·4 months	3·9 months	N/A
**Di Giacomo 2011 (141)**	P	27	Italy.	Skin cancer	55	14	Melanoma	N/A	N/A	N/A	ipilimumab	CTLA-4	8.5 months	9.6 months	29 months	N/A
**Royal 2010 (142)**	P	27	USA	Pancreatic cancer	55	15	ADC.	24	12	20	ipilimumab	CTLA-4	N/A	10.9 months	N/A	N/A
**Ralph 2010 (143)**	P	18	UK	Gastric/esophageal	56	14	ADC	N/A	N/A	18	tremelimumab	CTLA-4	N/A	Median survival for [illegible]	N/A	N/A
**Yang 2007 (144)**	P	40	USA	Renal cancer	N/A	28	RCC	39	N/A	2	ipilimumab	CTLA-4	N/A	N/A	N/A	17
**Maker 2006 (145)**	P	46	USA	Skin Cancer	48	32	Melanoma	46	14	28	ipilimumab	CTLA-4	N/A	N/A	N/A	N/A
**Reuben 2006 (146)**	P	30	USA	Skin Cancer	58	19	Melanoma	2	11	17	ticilimumab	CTLA-4	N/A	N/A	N/A	N/A
**Maker 2005 (147)**	P	36	USA	Skin Cancer	47.1	22	Melanoma	36	7	8	ipilimumab	CTLA-4	N/A	N/A	N/A	N/A

ACC: adenoid cystic carcinoma ADC: Adenocarcinoma, CTH: chemotherapy, HEENT: Head, Eyes, Ears, Nose, & Throat, HNSCC: Head & Neck squamous cell carcinoma, HCC: hepatocellular carcinoma, NSCLC: Non-small cell lung cancer, NOS: Not Otherwise Specified, P: Prospective, RCT: Randomized Controlled Trial, R: Retrospective SCC: squamous cell carcinoma, TC: thymic carcinoma, UC: Urothelial carcinoma.

Supplementary Table S4: Summary of criteria of different variables included in meta-analysis

	Average	SD	Median	Min	Max
**Study period (years)**	1.75	1.16	1.42	0.25	7.33
**Age**	59.22	8.44	60.75	19	72
**Males (%)**	47.64	-	-	0	100
**Underwent surgery (%)**	31.2	-	-	0	100
**Underwent radiotherapy (%)**	17.2	-	-	0	100
**Underwent chemotherapy (%)**	16.15	-	-	0	100

Supplementary Table S5: List of the 45 excluded studies for the sensitivity analysis

Aamdal 2021	Awada 2020	Bjoern 2016	Chalmers 2019	Chang 2021
Chu 2021	Chung 2019	DAngelo 2017	Da Gama Duarte 2018	Di Giacomo 2019
Duffy 2017	Graff 2020	Graff 2016	Gray 2019	Haag 2018
Hodi 2014	Kawazoe 2020	Kelly 2020	Kim 2020	Kitano 2021
Lam 2021	Le 2013	Liu 2020	Madan 2012	Mahalingam 2020
Maker 2005	Massarelli 2019	ONeill 2020	Ray 2016	Reilley 2017
Rodriguez 2020	Rozeman 2020	Sahebjam 2021	Sakamuri 2018	Shenderov 2021
Thomas 2019	Weber 2013	Weide 2017	Wilgenhof 2016	Wilky 2019
Xie 2020	Yarchoan 2020	Yuan 2021	Zamarin 2020	Zimmer 2019

*Studies in gray were added to the first 33 excluded studies (in white)

Supplementary Table S6: Subgroup analysis of pooled events rates in A) adverse events (AE) 3-4 and B) AE 5 by ITH drugs

A) AE 3-4				B) AE 5			
**ITH drug**	**N. Studies**	**PER (95% CI)**	**Subgroup difference**	**ITH drug**	**N. Studies**	**PER (95% CI)**	**Subgroup difference**
**Pembrolizumab**	**33**	**20.73% (13.92-29.71) ¶**	**p=0.1160**	**Pembrolizumab**	**38**	**2.90% (1.96-4.29) ¶¶**	**p=0.9798**
**Ipilimumab**	**13**	**42.20% (31.14-54.12) ¶**		**Ipilimumab**	**24**	**2.50% (1.47-4.23) ¶¶**	
**Nivolumab**	**5**	**21.92% (4.80-60.99)**		**Nivolumab**	**10**	**2.95% (0.98-8.56)**	
**Atezolizumab**	**3**	**42.40% (5.44-90.41)**		**Atezolizumab**	**3**	**2.54% (0.63-9.61)**	
**Toripalimab**	**3**	**10.78% (0.32-82.18)**		**Camrelizumab**	**3**	**1.49% (0.30-7.04)**	
**Avelumab**	**2**	**21.94% (2.80-73.28)**		**Durvalumab**	**3**	**1.07% (0.22-5.12)**	
**Camrelizumab**	**2**	**46.87% (11.70-85.46)**		**Tremelimumab**	**3**	**3.02% (0.75-11.34)**	
**Durvalumab**	**2**	**24.32% (14.30-38.24)**		**Avelumab**	**2**	**2.22% (0.31-14.28)**	
**Sintilimab**	**2**	**27.14% (3.49-79.31)**		**Sintilimab**	**2**	**4.84% (1.57-13.97)**	
**Cemiplimab**	**1**	**30.77% (12.04-59.07)**		**Toripalimab**	**2**	**1.51% (0.21-9.97)**	
**M7824**	**1**	**21.05% (8.13-44.55)**		**Cemiplimab**	**1**	**3.57% (0.22-38.39)**	
**Tremelimumab**	**1**	**6.9% (1.73-23.75)**		**M7824**	**1**	**2.50% (0.15-29.81)**	

¶ Subgroup analysis of AE 3-4 of Pembrolizumab and Ipilimumab was p=0.0030
¶¶ Subgroup analysis of AE 5 of Pembrolizumab and Ipilimumab was p=0.6563
AE: adverse events, PER: pooled events rate

Supplementary Table S7: Subgroup analysis of pooled events rates A) adverse events (AE) 3-4 and B) AE 5 by ITH class

	**A) AE 3-4**			**B) AE 5**		
**ITH drug**	**N. Studies**	**PER (95% CI)**	**Subgroup difference**	**N. Studies**	**PER (95% CI)**	**Subgroup difference**
**Anti PD-1**	46	22.16% (15.81-30.15)	p=0.0610	56	3.17% (2.31-4.35)	p=0.4817
**Anti PD-L1**	8	30.15% (14.43-52.49)		9	1.90% (0.79-4.49)	
**Anti CTLA-4**	14	38.64% (27.39-51.26)		27	2.56% (1.56-4.18)	

AE: adverse events, CTL A-4: cytotoxic T lymphocyte antigen 4, PD-1: programmed cell death 1 and PD-L1: programmed cell death 1 ligand 1, PER: pooled events rate

Supplementary Table S8: Subgroup analysis of primary outcomes on lung cancer only vs pancreatic cancer only vs all other cancers

Outcome	Group	Patients	Studies	PER (95%CI)	Heterogeneity (I^2, p-value)	Subgroup, p-value
**Any grade irAE**	Lung	545	17	74.81% [61.90; 84.45]	83.8%, p<0.0001	**0.0113**
	Pancreatic	97	4	96.19% [87.59; 98.90]	0%, p=0.5642	
	Others	1341	44	79.48% [70.03; 86.52]	87.1%, p<0.0001	
**Grade 3-4 irAE**	Lung	762	22	18.26% [11.13; 28.48]	84.5%, p<0.0001	**0.0207**
	Pancreatic	118	5	53.17% [28.56; 76.33]	81.0%, p=0.0003	
	Others	1414	49	29.48% [23.05; 36.83]	81.1%, p<0.0001	
**Grade 5 irAE**	Lung	734	22	2.38% [1.43; 3.95]	0%, p=0.9995	0.9134
	Pancreatic	315	7	3.28% [0.80; 12.50]	61.3%, p=0.0166	
	Others	2227	71	2.51% [1.85; 3.39]	0%, p=0.9996	
**Cardiac-related irAE**	Lung	961	29	2.76% [1.58; 4.77]	34.2%, p=0.0383	0.6213
	Pancreatic	345	8	1.73% [0.65; 4.53]	0%, p=0.8948	
	Others	3004	95	2.85% [2.19; 3.72]	7.6%, p=0.2758	
**Pulmonary-related irAE**	Lung	961	29	7.42% [5.17; 10.53]	41.1%, p=0.0120	**0.0059**
	Pancreatic	345	8	1.91% [0.80; 4.52]	0%, p=0.8980	
	Others	3004	95	4.53% [3.71; 5.52]	0%, p=0.5814	

References

(1) Lam, T.; Tsang, K.; Choi, H.; Lee, V.; Lam, K.; Chiang, C.; So, T.; Chan, W.; Nyaw, S.; Lim, F. Combination atezolizumab, bevacizumab, pemetrexed and carboplatin for metastatic EGFR mutated NSCLC after TKI failure. *Lung Cancer* **2021**, *159*, 18-26.
(2) Edenfield, W. J.; Chung, K.; O'Rourke, M.; Cull, E.; Martin, J.; Bowers, H.; Smith, W.; Gluck, W. L. A phase II study of durvalumab in combination with tremelimumab in patients with rare cancers. *The Oncologist* **2021**, *26* (9), e1499-e1507.
(3) Ferrarotto, R.; Sousa, L. G.; Qing, Y.; Kaya, D.; Stephen, B.; Jain, D.; Bell, D.; Pant, S.; Tsimberidou, A. M.; Janku, F. Pembrolizumab in patients with refractory cutaneous squamous cell carcinoma: a phase II trial. *Advances in Therapy* **2021**, *38* (8), 4581-4591.
(4) Zhu, X.; Cao, Y.; Liu, W. Stereotactic body radiotherapy plus pembrolizumab and trametinib versus stereotactic body radiotherapy plus gemcitabine for locally recurrent pancreatic cancer after surgical resection: an open-label, randomised, controlled, phase 2 trial (Retraction of Vol 22, Pg 1093, 2021). ELSEVIER SCIENCE INC STE 800, 230 PARK AVE, NEW YORK, NY 10169 USA: 2022.
(5) Reschke, R.; Gussek, P.; Boldt, A.; Sack, U.; Köhl, U.; Lordick, F.; Gora, T.; Kreuz, M.; Reiche, K.; Simon, J.-C. Distinct immune signatures indicative of treatment response and immune-related adverse events in melanoma patients under immune checkpoint inhibitor therapy. *International journal of molecular sciences* **2021**, *22* (15), 8017.
(6) Chang, K.-Y.; Chiang, N.-J.; Wu, S.-Y.; Yen, C.-J.; Chen, S.-H.; Yeh, Y.-M.; Li, C.-F.; Feng, X.; Wu, K.; Johnston, A. Phase 1b study of pegylated arginine deiminase (ADI-PEG 20) plus Pembrolizumab in advanced solid cancers. *Oncoimmunology* **2021**, *10* (1), 1943253.
(7) Zhang, W.; Yan, C.; Gao, X.; Li, X.; Cao, F.; Zhao, G.; Zhao, J.; Er, P.; Zhang, T.; Chen, X. Safety and feasibility of radiotherapy plus camrelizumab for locally advanced esophageal squamous cell carcinoma. *The oncologist* **2021**, *26* (7), e1110-e1124.
(8) Tsimberidou, A.-M.; Vo, H. H.; Subbiah, V.; Janku, F.; Piha-Paul, S.; Yilmaz, B.; Gong, J.; Naqvi, M. F.; Tu, S.-M.; Campbell, M. Pembrolizumab in patients with advanced metastatic germ cell tumors. *The Oncologist* **2021**, *26* (7), 558-e1098.
(9) Marcq, G.; Souhami, L.; Cury, F. L.; Salimi, A.; Aprikian, A.; Tanguay, S.; Vanhuyse, M.; Rajan, R.; Brimo, F.; Mansure, J. J. Phase 1 trial of atezolizumab plus trimodal therapy in patients with localized muscle-invasive bladder cancer. *International Journal of Radiation Oncology* Biology* Physics* **2021**, *110* (3), 738-741.
(10) Altorki, N. K.; McGraw, T. E.; Borczuk, A. C.; Saxena, A.; Port, J. L.; Stiles, B. M.; Lee, B. E.; Sanfilippo, N. J.; Scheff, R. J.; Pua, B. B. Neoadjuvant durvalumab with or without stereotactic body radiotherapy in patients with early-stage non-small-cell lung cancer: a single-centre, randomised phase 2 trial. *The Lancet Oncology* **2021**, *22* (6), 824-835.
(11) Aamdal, E.; Inderberg, E. M.; Ellingsen, E. B.; Rasch, W.; Brunsvig, P. F.; Aamdal, S.; Heintz, K.-M.; Vodák, D.; Nakken, S.; Hovig, E. Combining a universal telomerase based cancer vaccine with ipilimumab in patients with metastatic melanoma-five-year follow up of a phase I/IIa trial. *Frontiers in Immunology* **2021**, *12*, 663865.
(12) Di Noia, V.; D'Argento, E.; Pilotto, S.; Vita, E.; Ferrara, M. G.; Damiano, P.; Ribelli, M.; Cannella, A.; Virtuoso, A.; Fattorossi, A. Blood serum amyloid A as potential biomarker of pembrolizumab efficacy for patients affected by advanced non-small cell lung cancer overexpressing PD-L1: results of the exploratory "FoRECATT" study. *Cancer Immunology, Immunotherapy* **2021**, *70*, 1583-1592.
(13) Shenderov, E.; Boudadi, K.; Fu, W.; Wang, H.; Sullivan, R.; Jordan, A.; Dowling, D.; Harb, R.; Schonhoft, J.; Jendrisak, A. Nivolumab plus ipilimumab, with or without enzalutamide, in AR-V7-expressing metastatic castration-resistant prostate cancer: A phase-2 nonrandomized clinical trial. *The Prostate* **2021**, *81* (6), 326-338.
(14) Sahebjam, S.; Forsyth, P. A.; Tran, N. D.; Arrington, J. A.; Macaulay, R.; Etame, A. B.; Walko, C. M.; Boyle, T.; Peguero, E. N.; Jaglal, M. Hypofractionated stereotactic re-irradiation with pembrolizumab and bevacizumab in patients with recurrent high-grade gliomas: results from a phase I study. *Neuro-oncology* **2021**, *23* (4), 677-686.
(15) Chu, T.; Zhong, R.; Zhong, H.; Zhang, B.; Zhang, W.; Shi, C.; Qian, J.; Zhang, Y.; Chang, Q.; Zhang, X. Phase 1b study of sintilimab plus anlotinib as first-line therapy in patients with advanced NSCLC. *Journal of Thoracic Oncology* **2021**, *16* (4), 643-652.

(16) Ma, Y.; Fang, W.; Zhao, H.; Bathena, S. P.; Tendolkar, A.; Sheng, J.; Zhang, L. A Phase I Dose Escalation Study of the Safety, Tolerability, and Pharmacokinetics of Ipilimumab in Chinese Patients with Select Advanced Solid Tumors. *The Oncologist* **2021**, *26* (4), e549-e566.
(17) Cascone, T.; William Jr, W. N.; Weissferdt, A.; Leung, C. H.; Lin, H. Y.; Pataer, A.; Godoy, M. C.; Carter, B. W.; Federico, L.; Reuben, A. Neoadjuvant nivolumab or nivolumab plus ipilimumab in operable non-small cell lung cancer: the phase 2 randomized NEOSTAR trial. *Nature medicine* **2021**, *27* (3), 504-514.
(18) Eichhorn, F.; Klotz, L. V.; Kriegsmann, M.; Bischoff, H.; Schneider, M. A.; Muley, T.; Kriegsmann, K.; Haberkorn, U.; Heussel, C. P.; Savai, R. Neoadjuvant anti-programmed death-1 immunotherapy by pembrolizumab in resectable non-small cell lung cancer: First clinical experience. *Lung Cancer* **2021**, *153*, 150-157.
(19) Pelster, M. S.; Gruschkus, S. K.; Bassett, R.; Gombos, D. S.; Shephard, M.; Posada, L.; Glover, M. S.; Simien, R.; Diab, A.; Hwu, P. Nivolumab and ipilimumab in metastatic uveal melanoma: results from a single-arm phase II study. *Journal of Clinical Oncology* **2021**, *39* (6), 599.
(20) Sarfaty, M.; Whiting, K.; Teo, M. Y.; Lee, C. H.; Peters, V.; Durocher, J.; Regazzi, A. M.; McCoy, A. S.; Hettich, G.; Jungbluth, A. A. A phase II trial of durvalumab and tremelimumab in metastatic, non-urothelial carcinoma of the urinary tract. *Cancer Medicine* **2021**, *10* (3), 1074-1083.
(21) Yuan, Y.; Lee, J. S.; Yost, S. E.; Frankel, P. H.; Ruel, C.; Egelston, C. A.; Guo, W.; Gillece, J. D.; Folkerts, M.; Reining, L. A phase II clinical trial of pembrolizumab and enobosarm in patients with androgen receptor-positive metastatic triple-negative breast cancer. *The Oncologist* **2021**, *26* (2), 99-e217.
(22) Perez, B. A.; Kim, S.; Wang, M.; Karimi, A. M.; Powell, C.; Li, J.; Dilling, T. J.; Chiappori, A.; Latifi, K.; Rose, T. Prospective single-arm phase 1 and 2 study: ipilimumab and nivolumab with thoracic radiation therapy after platinum chemotherapy in extensive-stage small cell lung cancer. *International Journal of Radiation Oncology* Biology* Physics* **2021**, *109* (2), 425-435.
(23) Maity, A.; Mick, R.; Rengan, R.; Mitchell, T. C.; Amaravadi, R. K.; Schuchter, L. M.; Pryma, D. A.; Patsch, D. M.; Maity, A. P.; Minn, A. J. A stratified phase I dose escalation trial of hypofractionated radiotherapy followed by ipilimumab in metastatic melanoma: long-term follow-up and final outcomes. *Oncoimmunology* **2021**, *10* (1), 1863631.
(24) Kitano, S.; Shimizu, T.; Koyama, T.; Ebata, T.; Iwasa, S.; Kondo, S.; Shimomura, A.; Fujiwara, Y.; Yamamoto, N.; Paccaly, A. Dose exploration results from Phase 1 study of cemiplimab, a human monoclonal programmed death (PD)-1 antibody, in Japanese patients with advanced malignancies. *Cancer Chemotherapy and Pharmacology* **2021**, *87*, 53-64.
(25) Mahmood, U.; Bang, A.; Chen, Y.-H.; Mak, R. H.; Lorch, J. H.; Hanna, G. J.; Nishino, M.; Manuszak, C.; Thrash, E. M.; Severgnini, M. A randomized phase 2 study of pembrolizumab with or without radiation in patients with recurrent or metastatic adenoid cystic carcinoma. *International Journal of Radiation Oncology* Biology* Physics* **2021**, *109* (1), 134-144.
(26) Pakkala, S.; Higgins, K.; Chen, Z.; Sica, G.; Steuer, C.; Zhang, C.; Zhang, G.; Wang, S.; Hossain, M. S.; Nazha, B. Durvalumab and tremelimumab with or without stereotactic body radiation therapy in relapsed small cell lung cancer: a randomized phase II study. *Journal for Immunotherapy of Cancer* **2020**, *8* (2).
(27) Majd, N.; Waguespack, S. G.; Janku, F.; Fu, S.; Penas-Prado, M.; Xu, M.; Alshawa, A.; Kamiya-Matsuoka, C.; Raza, S. M.; McCutcheon, I. E. Efficacy of pembrolizumab in patients with pituitary carcinoma: report of four cases from a phase II study. *Journal for ImmunoTherapy of Cancer* **2020**, *8* (2).
(28) van Dijk, N.; Gil-Jimenez, A.; Silina, K.; Hendricksen, K.; Smit, L. A.; de Feijter, J. M.; van Montfoort, M. L.; van Rooijen, C.; Peters, D.; Broeks, A. Preoperative ipilimumab plus nivolumab in locoregionally advanced urothelial cancer: the NABUCCO trial. *Nature medicine* **2020**, *26* (12), 1839-1844.
(29) Gao, J.; Navai, N.; Alhalabi, O.; Siefker-Radtke, A.; Campbell, M. T.; Tidwell, R. S.; Guo, C. C.; Kamat, A. M.; Matin, S. F.; Araujo, J. C. Neoadjuvant PD-L1 plus CTLA-4 blockade in patients with cisplatin-ineligible operable high-risk urothelial carcinoma. *Nature medicine* **2020**, *26* (12), 1845-1851.
(30) Welsh, J. W.; Heymach, J. V.; Guo, C.; Menon, H.; Klein, K.; Cushman, T. R.; Verma, V.; Hess, K. R.; Shroff, G.; Tang, C. Phase 1/2 trial of pembrolizumab and concurrent chemoradiation therapy for limited-stage SCLC. *Journal of Thoracic Oncology* **2020**, *15* (12), 1919-1927.
(31) Duska, L. R.; Scalici, J. M.; Temkin, S. M.; Schwarz, J. K.; Crane, E. K.; Moxley, K. M.; Hamilton, C. A.; Wethington, S. L.; Petroni, G. R.; Varhegyi, N. E. Results of an early safety analysis of a study of the combination of pembrolizumab and pelvic chemoradiation in locally advanced cervical cancer. *Cancer* **2020**, *126* (22), 4948-4956.

(32) Schweizer, C.; Schubert, P.; Rutzner, S.; Eckstein, M.; Haderlein, M.; Lettmaier, S.; Semrau, S.; Gostian, A.-O.; Frey, B.; Gaipl, U. S. Prospective evaluation of the prognostic value of immune-related adverse events in patients with non-melanoma solid tumour treated with PD-1/PD-L1 inhibitors alone and in combination with radiotherapy. *European Journal of Cancer* **2020**, *140*, 55-62.
(33) Takahashi, A.; Namikawa, K.; Ogata, D.; Nakano, E.; Jinnai, S.; Nakama, K.; Tsutsui, K.; Muto, Y.; Mizuta, H.; Yamazaki, N. Real-world efficacy and safety data of nivolumab and ipilimumab combination therapy in Japanese patients with advanced melanoma. *The Journal of Dermatology* **2020**, *47* (11), 1267-1275.
(34) Awada, G.; Salama, L. B.; De Cremer, J.; Schwarze, J. K.; Fischbuch, L.; Seynaeve, L.; Du Four, S.; Vanbinst, A.-M.; Michotte, A.; Everaert, H. Axitinib plus avelumab in the treatment of recurrent glioblastoma: a stratified, open-label, single-center phase 2 clinical trial (GliAvAx). *Journal for immunotherapy of cancer* **2020**, *8* (2).
(35) Welsh, J.; Menon, H.; Chen, D.; Verma, V.; Tang, C.; Altan, M.; Hess, K.; De Groot, P.; Nguyen, Q.-N.; Varghese, R. Pembrolizumab with or without radiation therapy for metastatic non-small cell lung cancer: a randomized phase I/II trial. *Journal for immunotherapy of cancer* **2020**, *8* (2).
(36) Wang, Z.; Ying, J.; Xu, J.; Yuan, P.; Duan, J.; Bai, H.; Guo, C.; Li, L.; Yang, Z.; Wan, R. Safety, Antitumor Activity, and Pharmacokinetics of Toripalimab, a Programmed Cell Death 1 Inhibitor, in Patients With Advanced Non–Small Cell Lung Cancer: A Phase 1 Trial. *JAMA network open* **2020**, *3* (10), e2013770-e2013770.
(37) Schoenfeld, J. D.; Hanna, G. J.; Jo, V. Y.; Rawal, B.; Chen, Y.-H.; Catalano, P. S.; Lako, A.; Ciantra, Z.; Weirather, J. L.; Criscitiello, S. Neoadjuvant nivolumab or nivolumab plus ipilimumab in untreated oral cavity squamous cell carcinoma: a phase 2 open-label randomized clinical trial. *JAMA oncology* **2020**, *6* (10), 1563-1570.
(38) O'Neill, C.; Hayat, T.; Hamm, J.; Healey, M.; Zheng, Q.; Li, Y.; Martin II, R. C. A phase 1b trial of concurrent immunotherapy and irreversible electroporation in the treatment of locally advanced pancreatic adenocarcinoma. *Surgery* **2020**, *168* (4), 610-616.
(39) Frumovitz, M.; Westin, S. N.; Salvo, G.; Zarifa, A.; Xu, M.; Yap, T. A.; Rodon, A. J.; Karp, D. D.; Abonofal, A.; Jazaeri, A. A. Phase II study of pembrolizumab efficacy and safety in women with recurrent small cell neuroendocrine carcinoma of the lower genital tract. *Gynecologic oncology* **2020**, *158* (3), 570-575.
(40) Ratnayake, G.; Reinwald, S.; Shackleton, M.; Moore, M.; Voskoboynik, M.; Ruben, J.; van Zelm, M. C.; Yu, D.; Ward, R.; Smith, R. Stereotactic radiation therapy combined with immunotherapy against metastatic melanoma: long-term results of a phase 1 clinical trial. *International Journal of Radiation Oncology* Biology* Physics* **2020**, *108* (1), 150-156.
(41) Boutros, C.; Chaput-Gras, N.; Lanoy, E.; Larive, A.; Mateus, C.; Routier, E.; Sun, R.; Tao, Y. G.; Massard, C.; Bahleda, R. Dose escalation phase 1 study of radiotherapy in combination with anti-cytotoxic-T-lymphocyte-associated antigen 4 monoclonal antibody ipilimumab in patients with metastatic melanoma. *Journal for Immunotherapy of Cancer* **2020**, *8* (2).
(42) Kawazoe, A.; Fukuoka, S.; Nakamura, Y.; Kuboki, Y.; Wakabayashi, M.; Nomura, S.; Mikamoto, Y.; Shima, H.; Fujishiro, N.; Higuchi, T. Lenvatinib plus pembrolizumab in patients with advanced gastric cancer in the first-line or second-line setting (EPOC1706): an open-label, single-arm, phase 2 trial. *The Lancet Oncology* **2020**, *21* (8), 1057-1065.
(43) Wei, X. L.; Ren, C.; Wang, F. H.; Zhang, Y.; Zhao, H. Y.; Zou, B. Y.; Wang, Z. Q.; Qiu, M. Z.; Zhang, D. S.; Luo, H. Y. A phase I study of toripalimab, an anti-PD-1 antibody, in patients with refractory malignant solid tumors. *Cancer Communications* **2020**, *40* (8), 345-354.
(44) Brastianos, P. K.; Lee, E. Q.; Cohen, J. V.; Tolaney, S. M.; Lin, N. U.; Wang, N.; Chukwueke, U.; White, M. D.; Nayyar, N.; Kim, A. Single-arm, open-label phase 2 trial of pembrolizumab in patients with leptomeningeal carcinomatosis. *Nature medicine* **2020**, *26* (8), 1280-1284.
(45) Kim, C.; Liu, S. V.; Subramaniam, D. S.; Torres, T.; Loda, M.; Esposito, G.; Giaccone, G. Phase I study of the 177Lu-DOTA0-Tyr3-Octreotate (lutathera) in combination with nivolumab in patients with neuroendocrine tumors of the lung. *Journal for Immunotherapy of Cancer* **2020**, *8* (2).
(46) Graff, J. N.; Beer, T. M.; Alumkal, J. J.; Slottke, R. E.; Redmond, W. L.; Thomas, G. V.; Thompson, R. F.; Wood, M. A.; Koguchi, Y.; Chen, Y. A phase II single-arm study of pembrolizumab with enzalutamide in men with metastatic castration-resistant prostate cancer progressing on enzalutamide alone. *Journal for immunotherapy of cancer* **2020**, *8* (2).
(47) Ferrarotto, R.; Bell, D.; Rubin, M. L.; Hutcheson, K. A.; Johnson, J. M.; Goepfert, R. P.; Phan, J.; Elamin, Y. Y.; Torman, D. K.; Warneke, C. L. Impact of neoadjuvant durvalumab with or without tremelimumab on CD8+ tumor lymphocyte density, safety, and efficacy in patients with oropharynx cancer: CIAO trial results. *Clinical Cancer Research* **2020**, *26* (13), 3211-3219.

(48) Postow, M. A.; Knox, S. J.; Goldman, D. A.; Elhanati, Y.; Mavinkurve, V.; Wong, P.; Halpenny, D.; Reddy, S. K.; Vado, K.; McCabe, D. A prospective, phase 1 trial of nivolumab, ipilimumab, and radiotherapy in patients with advanced melanoma. *Clinical Cancer Research* **2020**, *26* (13), 3193-3201.
(49) Zamarin, D.; Walderich, S.; Holland, A.; Zhou, Q.; Iasonos, A. E.; Torrisi, J. M.; Merghoub, T.; Chesebrough, L. F.; Mcdonnell, A. S.; Gallagher, J. M. Safety, immunogenicity, and clinical efficacy of durvalumab in combination with folate receptor alpha vaccine TPIV200 in patients with advanced ovarian cancer: a phase II trial. *Journal for ImmunoTherapy of Cancer* **2020**, *8* (1).
(50) Feng, K.; Liu, Y.; Zhao, Y.; Yang, Q.; Dong, L.; Liu, J.; Li, X.; Zhao, Z.; Mei, Q.; Han, W. Efficacy and biomarker analysis of nivolumab plus gemcitabine and cisplatin in patients with unresectable or metastatic biliary tract cancers: results from a phase II study. *Journal for immunotherapy of cancer* **2020**, *8* (1).
(51) Kanda, S.; Ohe, Y.; Goto, Y.; Horinouchi, H.; Fujiwara, Y.; Nokihara, H.; Yamamoto, N.; Yamamoto, T.; Tamura, T. Five-year safety and efficacy data from a phase Ib study of nivolumab and chemotherapy in advanced non-small-cell lung cancer. *Cancer Science* **2020**, *111* (6), 1933-1942.
(52) Arrieta, O.; Barrón, F.; Ramírez-Tirado, L. A.; Zatarain-Barrón, Z. L.; Cardona, A. F.; Díaz-García, D.; Ramos, M. Y.; Mota-Vega, B.; Carmona, A.; Álvarez, M. P. P. Efficacy and safety of pembrolizumab plus docetaxel vs docetaxel alone in patients with previously treated advanced non–small cell lung cancer: The PROLUNG phase 2 randomized clinical trial. *JAMA oncology* **2020**, *6* (6), 856-864.
(53) Rozeman, E. A.; Prevoo, W.; Meier, M. A.; Sikorska, K.; Van, T. M.; van de Wiel, B. A.; van Der Wal, J. E.; Mallo, H. A.; Grijpink-Ongering, L. G.; Broeks, A. Phase Ib/II trial testing combined radiofrequency ablation and ipilimumab in uveal melanoma (SECIRA-UM). *Melanoma Research* **2020**, *30* (3), 252-260.
(54) Xie, C.; Duffy, A. G.; Brar, G.; Fioravanti, S.; Mabry-Hrones, D.; Walker, M.; Bonilla, C. M.; Wood, B. J.; Citrin, D. E.; Gil Ramirez, E. M. Immune checkpoint blockade in combination with stereotactic body radiotherapy in patients with metastatic pancreatic ductal adenocarcinoma. *Clinical Cancer Research* **2020**, *26* (10), 2318-2326.
(55) Music, M.; Iafolla, M.; Soosaipillai, A.; Batruch, I.; Prassas, I.; Pintilie, M.; Hansen, A. R.; Bedard, P. L.; Lheureux, S.; Spreafico, A. Predicting response and toxicity to PD-1 inhibition using serum autoantibodies identified from immuno-mass spectrometry. *F1000Research* **2020**, *9*.
(56) Liu, J.; Liu, Q.; Li, Y.; Li, Q.; Su, F.; Yao, H.; Su, S.; Wang, Q.; Jin, L.; Wang, Y. Efficacy and safety of camrelizumab combined with apatinib in advanced triple-negative breast cancer: an open-label phase II trial. *Journal for immunotherapy of cancer* **2020**, *8* (1).
(57) Xie, L.; Xu, J.; Sun, X.; Guo, W.; Gu, J.; Liu, K.; Zheng, B.; Ren, T.; Huang, Y.; Tang, X. Apatinib plus camrelizumab (anti-PD1 therapy, SHR-1210) for advanced osteosarcoma (APFAO) progressing after chemotherapy: a single-arm, open-label, phase 2 trial. *Journal for immunotherapy of cancer* **2020**, *8* (1).
(58) Goldberg, S. B.; Schalper, K. A.; Gettinger, S. N.; Mahajan, A.; Herbst, R. S.; Chiang, A. C.; Lilenbaum, R.; Wilson, F. H.; Omay, S. B.; James, B. Y. Pembrolizumab for management of patients with NSCLC and brain metastases: long-term results and biomarker analysis from a non-randomised, open-label, phase 2 trial. *The Lancet Oncology* **2020**, *21* (5), 655-663.
(59) Gao, S.; Li, N.; Gao, S.; Xue, Q.; Ying, J.; Wang, S.; Tao, X.; Zhao, J.; Mao, Y.; Wang, B. Neoadjuvant PD-1 inhibitor (Sintilimab) in NSCLC. *Journal of Thoracic Oncology* **2020**, *15* (5), 816-826.
(60) Kamath, S. D.; Kalyan, A.; Kircher, S.; Nimeiri, H.; Fought, A. J.; Benson III, A.; Mulcahy, M. Ipilimumab and gemcitabine for advanced pancreatic cancer: a phase Ib study. *The oncologist* **2020**, *25* (5), e808-e815.
(61) Naing, A.; Meric-Bernstam, F.; Stephen, B.; Karp, D. D.; Hajjar, J.; Ahnert, J. R.; Piha-Paul, S. A.; Colen, R. R.; Jimenez, C.; Raghav, K. P. Phase 2 study of pembrolizumab in patients with advanced rare cancers. *Journal for immunotherapy of cancer* **2020**, *8* (1).
(62) Kelly, C. M.; Antonescu, C. R.; Bowler, T.; Munhoz, R.; Chi, P.; Dickson, M. A.; Gounder, M. M.; Keohan, M. L.; Movva, S.; Dholakia, R. Objective response rate among patients with locally advanced or metastatic sarcoma treated with talimogene laherparepvec in combination with pembrolizumab: a phase 2 clinical trial. *JAMA oncology* **2020**, *6* (3), 402-408.
(63) Ross, A. E.; Hurley, P. J.; Tran, P. T.; Rowe, S. P.; Benzon, B.; Neal, T. O.; Chapman, C.; Harb, R.; Milman, Y.; Trock, B. J. A pilot trial of pembrolizumab plus prostatic cryotherapy for men with newly diagnosed oligometastatic hormone-sensitive prostate cancer. *Prostate cancer and prostatic diseases* **2020**, *23* (1), 184-193.

(64) Rodriguez, C. P.; Wu, Q.; Voutsinas, J.; Fromm, J. R.; Jiang, X.; Pillarisetty, V. G.; Lee, S. M.; Santana-Davila, R.; Goulart, B.; Baik, C. S. A phase II trial of pembrolizumab and vorinostat in recurrent metastatic head and neck squamous cell carcinomas and salivary gland cancer. *Clinical Cancer Research* **2020**, *26* (4), 837-845.
(65) Shah, A. N.; Flaum, L.; Helenowski, I.; Santa-Maria, C. A.; Jain, S.; Rademaker, A.; Nelson, V.; Tsarwhas, D.; Cristofanilli, M.; Gradishar, W. Phase II study of pembrolizumab and capecitabine for triple negative and hormone receptor-positive, HER2− negative endocrine-refractory metastatic breast cancer. *Journal for immunotherapy of cancer* **2020**, *8* (1).
(66) Yarchoan, M.; Huang, C. Y.; Zhu, Q.; Ferguson, A. K.; Durham, J. N.; Anders, R. A.; Thompson, E. D.; Rozich, N. S.; Thomas, D. L.; Nauroth, J. M. A phase 2 study of GVAX colon vaccine with cyclophosphamide and pembrolizumab in patients with mismatch repair proficient advanced colorectal cancer. *Cancer medicine* **2020**, *9* (4), 1485-1494.
(67) Lin, S. H.; Lin, Y.; Yao, L.; Kalhor, N.; Carter, B. W.; Altan, M.; Blumenschein, G.; Byers, L. A.; Fossella, F.; Gibbons, D. L. Phase II trial of concurrent atezolizumab with chemoradiation for unresectable NSCLC. *Journal of Thoracic Oncology* **2020**, *15* (2), 248-257.
(68) Welsh, J. W.; Heymach, J. V.; Chen, D.; Verma, V.; Cushman, T. R.; Hess, K. R.; Shroff, G.; Tang, C.; Skoulidis, F.; Jeter, M. Phase I trial of pembrolizumab and radiation therapy after induction chemotherapy for extensive-stage small cell lung cancer. *Journal of Thoracic Oncology* **2020**, *15* (2), 266-273.
(69) Mahalingam, D.; Wilkinson, G. A.; Eng, K. H.; Fields, P.; Raber, P.; Moseley, J. L.; Cheetham, K.; Coffey, M.; Nuovo, G.; Kalinski, P. Pembrolizumab in combination with the oncolytic virus pelareorep and chemotherapy in patients with advanced pancreatic adenocarcinoma: a phase Ib study. *Clinical Cancer Research* **2020**, *26* (1), 71-81.
(70) Elbers, J. B.; Al-Mamgani, A.; Tesseslaar, M. E.; van den Brekel, M. W.; Lange, C. A.; van der Wal, J. E.; Verheij, M.; Zuur, C. L.; de Boer, J. P. Immuno-radiotherapy with cetuximab and avelumab for advanced stage head and neck squamous cell carcinoma: Results from a phase-I trial. *Radiotherapy and Oncology* **2020**, *142*, 79-84.
(71) Di Giacomo, A. M.; Covre, A.; Finotello, F.; Rieder, D.; Danielli, R.; Sigalotti, L.; Giannarelli, D.; Petitprez, F.; Lacroix, L.; Valente, M. Guadecitabine plus ipilimumab in unresectable melanoma: the NIBIT-M4 clinical trial. *Clinical Cancer Research* **2019**, *25* (24), 7351-7362.
(72) Welsh, J. W.; Tang, C.; De Groot, P.; Naing, A.; Hess, K. R.; Heymach, J. V.; Papadimitrakopoulou, V. A.; Cushman, T. R.; Subbiah, V.; Chang, J. Y. Phase II trial of ipilimumab with stereotactic radiation therapy for metastatic disease: outcomes, toxicities, and low-dose radiation–related abscopal responses. *Cancer immunology research* **2019**, *7* (12), 1903-1909.
(73) Gray, J. E.; Saltos, A.; Tanvetyanon, T.; Haura, E. B.; Creelan, B.; Antonia, S. J.; Shafique, M.; Zheng, H.; Dai, W.; Saller, J. J. Phase I/Ib study of pembrolizumab plus vorinostat in advanced/metastatic non–small cell lung cancer. *Clinical Cancer Research* **2019**, *25* (22), 6623-6632.
(74) Qiao, G.; Wang, X.; Zhou, X.; Morse, M. A.; Wu, J.; Wang, S.; Song, Y.; Jiang, N.; Zhao, Y.; Zhou, L. Immune correlates of clinical benefit in a phase I study of hyperthermia with adoptive T cell immunotherapy in patients with solid tumors. *International Journal of Hyperthermia* **2019**, *36* (sup1), 74-82.
(75) Chintakuntlawar, A. V.; Yin, J.; Foote, R. L.; Kasperbauer, J. L.; Rivera, M.; Asmus, E.; Garces, N. I.; Janus, J. R.; Liu, M.; Ma, D. J. A phase 2 study of pembrolizumab combined with chemoradiotherapy as initial treatment for anaplastic thyroid cancer. *Thyroid* **2019**, *29* (11), 1615-1622.
(76) Feun, L. G.; Li, Y. Y.; Wu, C.; Wangpaichitr, M.; Jones, P. D.; Richman, S. P.; Madrazo, B.; Kwon, D.; Garcia-Buitrago, M.; Martin, P. Phase 2 study of pembrolizumab and circulating biomarkers to predict anticancer response in advanced, unresectable hepatocellular carcinoma. *Cancer* **2019**, *125* (20), 3603-3614.
(77) Habra, M. A.; Stephen, B.; Campbell, M.; Hess, K.; Tapia, C.; Xu, M.; Rodon Ahnert, J.; Jimenez, C.; Lee, J. E.; Perrier, N. D. Phase II clinical trial of pembrolizumab efficacy and safety in advanced adrenocortical carcinoma. *Journal for immunotherapy of cancer* **2019**, *7*, 1-9.
(78) Cho, J.; Kim, H. S.; Ku, B. M.; Choi, Y.-L.; Cristescu, R.; Han, J.; Sun, J.-M.; Lee, S.-H.; Ahn, J. S.; Park, K. Pembrolizumab for patients with refractory or relapsed thymic epithelial tumor: an open-label phase II trial. *J clin oncol* **2019**, *37* (24), 2162-2170.
(79) Chalmers, A. W.; Patel, S.; Boucher, K.; Cannon, L.; Esplin, M.; Luckart, J.; Graves, N.; Van Duren, T.; Akerley, W. Phase I trial of targeted EGFR or ALK therapy with ipilimumab in metastatic NSCLC with long-term follow-up. *Targeted Oncology* **2019**, *14*, 417-421.

(80) Mego, M.; Svetlovska, D.; Chovanec, M.; Rečkova, M.; Rejlekova, K.; Obertova, J.; Palacka, P.; Sycova-Mila, Z.; De Giorgi, U.; Mardiak, J. Phase II study of avelumab in multiple relapsed/refractory germ cell cancer. *Investigational new drugs* **2019**, *37*, 748-754.
(81) Thomas, A.; Vilimas, R.; Trindade, C.; Erwin-Cohen, R.; Roper, N.; Xi, L.; Krishnasamy, V.; Levy, E.; Mammen, A.; Nichols, S. Durvalumab in combination with olaparib in patients with relapsed SCLC: results from a phase II study. *Journal of Thoracic Oncology* **2019**, *14* (8), 1447-1457.
(82) Zimmer, A. S.; Nichols, E.; Cimino-Mathews, A.; Peer, C.; Cao, L.; Lee, M.-J.; Kohn, E. C.; Annunziata, C. M.; Lipkowitz, S.; Trepel, J. B. A phase I study of the PD-L1 inhibitor, durvalumab, in combination with a PARP inhibitor, olaparib, and a VEGFR1–3 inhibitor, cediranib, in recurrent women's cancers with biomarker analyses. *Journal for immunotherapy of cancer* **2019**, *7* (1), 1-8.
(83) Suresh, K.; Naidoo, J.; Zhong, Q.; Xiong, Y.; Mammen, J.; De Flores, M. V.; Cappelli, L.; Balaji, A.; Palmer, T.; Forde, P. M. The alveolar immune cell landscape is dysregulated in checkpoint inhibitor pneumonitis. *The Journal of clinical investigation* **2019**, *129* (10), 4305-4315.
(84) Sundahl, N.; Seremet, T.; Van Dorpe, J.; Neyns, B.; Ferdinande, L.; Meireson, A.; Brochez, L.; Kruse, V.; Ost, P. Phase 2 trial of nivolumab combined with stereotactic body radiation therapy in patients with metastatic or locally advanced inoperable melanoma. *International Journal of Radiation Oncology* Biology* Physics* **2019**, *104* (4), 828-835.
(85) Voorwerk, L.; Slagter, M.; Horlings, H. M.; Sikorska, K.; van de Vijver, K. K.; de Maaker, M.; Nederlof, I.; Kluin, R. J.; Warren, S.; Ong, S. Immune induction strategies in metastatic triple-negative breast cancer to enhance the sensitivity to PD-1 blockade: the TONIC trial. *Nature medicine* **2019**, *25* (6), 920-928.
(86) Wilky, B. A.; Trucco, M. M.; Subhawong, T. K.; Florou, V.; Park, W.; Kwon, D.; Wieder, E. D.; Kolonias, D.; Rosenberg, A. E.; Kerr, D. A. Axitinib plus pembrolizumab in patients with advanced sarcomas including alveolar soft-part sarcoma: a single-centre, single-arm, phase 2 trial. *The lancet oncology* **2019**, *20* (6), 837-848.
(87) Sundahl, N.; Vandekerkhove, G.; Decaestecker, K.; Meireson, A.; De Visschere, P.; Fonteyne, V.; De Maeseneer, D.; Reynders, D.; Goetghebeur, E.; Van Dorpe, J. Randomized phase 1 trial of pembrolizumab with sequential versus concomitant stereotactic body radiotherapy in metastatic urothelial carcinoma. *European urology* **2019**, *75* (5), 707-711.
(88) Disselhorst, M. J.; Quispel-Janssen, J.; Lalezari, F.; Monkhorst, K.; de Vries, J. F.; van der Noort, V.; Harms, E.; Burgers, S.; Baas, P. Ipilimumab and nivolumab in the treatment of recurrent malignant pleural mesothelioma (INITIATE): results of a prospective, single-arm, phase 2 trial. *The Lancet Respiratory Medicine* **2019**, *7* (3), 260-270.
(89) Huang, J.; Mo, H.; Zhang, W.; Chen, X.; Qu, D.; Wang, X.; Wu, D.; Wang, X.; Lan, B.; Yang, B. Promising efficacy of SHR-1210, a novel anti–programmed cell death 1 antibody, in patients with advanced gastric and gastroesophageal junction cancer in China. *Cancer* **2019**, *125* (5), 742-749.
(90) Chung, V.; Kos, F.; Hardwick, N.; Yuan, Y.; Chao, J.; Li, D.; Waisman, J.; Li, M.; Zurcher, K.; Frankel, P. Evaluation of safety and efficacy of p53MVA vaccine combined with pembrolizumab in patients with advanced solid cancers. *Clinical and Translational Oncology* **2019**, *21*, 363-372.
(91) Miyamoto, S.; Nomura, R.; Sato, K.; Awano, N.; Kuse, N.; Inomata, M.; Izumo, T.; Terada, Y.; Furuhata, Y.; Bae, Y. Nivolumab and stereotactic radiation therapy for the treatment of patients with Stage IV non-small-cell lung cancer. *Japanese journal of clinical oncology* **2019**, *49* (2), 160-164.
(92) Tang, B.; Yan, X.; Sheng, X.; Si, L.; Cui, C.; Kong, Y.; Mao, L.; Lian, B.; Bai, X.; Wang, X. Safety and clinical activity with an anti-PD-1 antibody JS001 in advanced melanoma or urologic cancer patients. *Journal of hematology & oncology* **2019**, *12*, 1-15.
(93) Ali, O. H.; Berner, F.; Bomze, D.; Fässler, M.; Diem, S.; Cozzio, A.; Jörger, M.; Früh, M.; Driessen, C.; Lenz, T. L. Human leukocyte antigen variation is associated with adverse events of checkpoint inhibitors. *European Journal of Cancer* **2019**, *107*, 8-14.
(94) Kluger, H. M.; Chiang, V.; Mahajan, A.; Zito, C. R.; Sznol, M.; Tran, T.; Weiss, S. A.; Cohen, J. V.; Yu, J.; Hegde, U. Long-term survival of patients with melanoma with active brain metastases treated with pembrolizumab on a phase II trial. *Journal of Clinical Oncology* **2019**, *37* (1), 52.
(95) Massarelli, E.; William, W.; Johnson, F.; Kies, M.; Ferrarotto, R.; Guo, M.; Feng, L.; Lee, J. J.; Tran, H.; Kim, Y. U. Combining immune checkpoint blockade and tumor-specific vaccine for patients with incurable human papillomavirus 16–related cancer: a phase 2 clinical trial. *JAMA oncology* **2019**, *5* (1), 67-73.

(96) Parikh, M.; Pan, C.-X.; Beckett, L. A.; Li, Y.; Robles, D. A.; Aujla, P. K.; Lara Jr, P. N. Pembrolizumab combined with either docetaxel or gemcitabine in patients with advanced or metastatic platinum-refractory urothelial cancer: results from a phase I study. *Clinical genitourinary cancer* **2018**, *16* (6), 421-428. e421.
(1) Lam, T.; Tsang, K.; Choi, H.; Lee, V.; Lam, K.; Chiang, C.; So, T.; Chan, W.; Nyaw, S.; Lim, F. Combination atezolizumab, bevacizumab, pemetrexed and carboplatin for metastatic EGFR mutated NSCLC after TKI failure. *Lung Cancer* **2021**, *159*, 18-26.
(2) Edenfield, W. J.; Chung, K.; O'Rourke, M.; Cull, E.; Martin, J.; Bowers, H.; Smith, W.; Gluck, W. L. A phase II study of durvalumab in combination with tremelimumab in patients with rare cancers. *The Oncologist* **2021**, *26* (9), e1499-e1507.
(3) Ferrarotto, R.; Sousa, L. G.; Qing, Y.; Kaya, D.; Stephen, B.; Jain, D.; Bell, D.; Pant, S.; Tsimberidou, A. M.; Janku, F. Pembrolizumab in patients with refractory cutaneous squamous cell carcinoma: a phase II trial. *Advances in Therapy* **2021**, *38* (8), 4581-4591.
(4) Zhu, X.; Cao, Y.; Liu, W. Stereotactic body radiotherapy plus pembrolizumab and trametinib versus stereotactic body radiotherapy plus gemcitabine for locally recurrent pancreatic cancer after surgical resection: an open-label, randomised, controlled, phase 2 trial (Retraction of Vol 22, Pg 1093, 2021). ELSEVIER SCIENCE INC STE 800, 230 PARK AVE, NEW YORK, NY 10169 USA: 2022.
(5) Reschke, R.; Gussek, P.; Boldt, A.; Sack, U.; Köhl, U.; Lordick, F.; Gora, T.; Kreuz, M.; Reiche, K.; Simon, J.-C. Distinct immune signatures indicative of treatment response and immune-related adverse events in melanoma patients under immune checkpoint inhibitor therapy. *International journal of molecular sciences* **2021**, *22* (15), 8017.
(6) Chang, K.-Y.; Chiang, N.-J.; Wu, S.-Y.; Yen, C.-J.; Chen, S.-H.; Yeh, Y.-M.; Li, C.-F.; Feng, X.; Wu, K.; Johnston, A. Phase 1b study of pegylated arginine deiminase (ADI-PEG 20) plus Pembrolizumab in advanced solid cancers. *Oncoimmunology* **2021**, *10* (1), 1943253.
(7) Zhang, W.; Yan, C.; Gao, X.; Li, X.; Cao, F.; Zhao, G.; Zhao, J.; Er, P.; Zhang, T.; Chen, X. Safety and feasibility of radiotherapy plus camrelizumab for locally advanced esophageal squamous cell carcinoma. *The oncologist* **2021**, *26* (7), e1110-e1124.
(8) Tsimberidou, A.-M.; Vo, H. H.; Subbiah, V.; Janku, F.; Piha-Paul, S.; Yilmaz, B.; Gong, J.; Naqvi, M. F.; Tu, S.-M.; Campbell, M. Pembrolizumab in patients with advanced metastatic germ cell tumors. *The Oncologist* **2021**, *26* (7), 558-e1098.
(9) Marcq, G.; Souhami, L.; Cury, F. L.; Salimi, A.; Aprikian, A.; Tanguay, S.; Vanhuyse, M.; Rajan, R.; Brimo, F.; Mansure, J. J. Phase 1 trial of atezolizumab plus trimodal therapy in patients with localized muscle-invasive bladder cancer. *International Journal of Radiation Oncology* Biology* Physics* **2021**, *110* (3), 738-741.
(10) Altorki, N. K.; McGraw, T. E.; Borczuk, A. C.; Saxena, A.; Port, J. L.; Stiles, B. M.; Lee, B. E.; Sanfilippo, N. J.; Scheff, R. J.; Pua, B. B. Neoadjuvant durvalumab with or without stereotactic body radiotherapy in patients with early-stage non-small-cell lung cancer: a single-centre, randomised phase 2 trial. *The Lancet Oncology* **2021**, *22* (6), 824-835.
(11) Aamdal, E.; Inderberg, E. M.; Ellingsen, E. B.; Rasch, W.; Brunsvig, P. F.; Aamdal, S.; Heintz, K.-M.; Vodák, D.; Nakken, S.; Hovig, E. Combining a universal telomerase based cancer vaccine with ipilimumab in patients with metastatic melanoma-five-year follow up of a phase I/IIa trial. *Frontiers in Immunology* **2021**, *12*, 663865.
(12) Di Noia, V.; D'Argento, E.; Pilotto, S.; Vita, E.; Ferrara, M. G.; Damiano, P.; Ribelli, M.; Cannella, A.; Virtuoso, A.; Fattorossi, A. Blood serum amyloid A as potential biomarker of pembrolizumab efficacy for patients affected by advanced non-small cell lung cancer overexpressing PD-L1: results of the exploratory "FoRECATT" study. *Cancer Immunology, Immunotherapy* **2021**, *70*, 1583-1592.
(13) Shenderov, E.; Boudadi, K.; Fu, W.; Wang, H.; Sullivan, R.; Jordan, A.; Dowling, D.; Harb, R.; Schonhoft, J.; Jendrisak, A. Nivolumab plus ipilimumab, with or without enzalutamide, in AR-V7-expressing metastatic castration-resistant prostate cancer: A phase-2 nonrandomized clinical trial. *The Prostate* **2021**, *81* (6), 326-338.
(14) Sahebjam, S.; Forsyth, P. A.; Tran, N. D.; Arrington, J. A.; Macaulay, R.; Etame, A. B.; Walko, C. M.; Boyle, T.; Peguero, E. N.; Jaglal, M. Hypofractionated stereotactic re-irradiation with pembrolizumab and bevacizumab in patients with recurrent high-grade gliomas: results from a phase I study. *Neuro-oncology* **2021**, *23* (4), 677-686.
(15) Chu, T.; Zhong, R.; Zhong, H.; Zhang, B.; Zhang, W.; Shi, C.; Qian, J.; Zhang, Y.; Chang, Q.; Zhang, X. Phase 1b study of sintilimab plus anlotinib as first-line therapy in patients with advanced NSCLC. *Journal of Thoracic Oncology* **2021**, *16* (4), 643-652.

(16) Ma, Y.; Fang, W.; Zhao, H.; Bathena, S. P.; Tendolkar, A.; Sheng, J.; Zhang, L. A Phase I Dose Escalation Study of the Safety, Tolerability, and Pharmacokinetics of Ipilimumab in Chinese Patients with Select Advanced Solid Tumors. *The Oncologist* **2021**, *26* (4), e549-e566.
(17) Cascone, T.; William Jr, W. N.; Weissferdt, A.; Leung, C. H.; Lin, H. Y.; Pataer, A.; Godoy, M. C.; Carter, B. W.; Federico, L.; Reuben, A. Neoadjuvant nivolumab or nivolumab plus ipilimumab in operable non-small cell lung cancer: the phase 2 randomized NEOSTAR trial. *Nature medicine* **2021**, *27* (3), 504-514.
(18) Eichhorn, F.; Klotz, L. V.; Kriegsmann, M.; Bischoff, H.; Schneider, M. A.; Muley, T.; Kriegsmann, K.; Haberkorn, U.; Heussel, C. P.; Savai, R. Neoadjuvant anti-programmed death-1 immunotherapy by pembrolizumab in resectable non-small cell lung cancer: First clinical experience. *Lung Cancer* **2021**, *153*, 150-157.
(19) Pelster, M. S.; Gruschkus, S. K.; Bassett, R.; Gombos, D. S.; Shephard, M.; Posada, L.; Glover, M. S.; Simien, R.; Diab, A.; Hwu, P. Nivolumab and ipilimumab in metastatic uveal melanoma: results from a single-arm phase II study. *Journal of Clinical Oncology* **2021**, *39* (6), 599.
(20) Sarfaty, M.; Whiting, K.; Teo, M. Y.; Lee, C. H.; Peters, V.; Durocher, J.; Regazzi, A. M.; McCoy, A. S.; Hettich, G.; Jungbluth, A. A. A phase II trial of durvalumab and tremelimumab in metastatic, non-urothelial carcinoma of the urinary tract. *Cancer Medicine* **2021**, *10* (3), 1074-1083.
(21) Yuan, Y.; Lee, J. S.; Yost, S. E.; Frankel, P. H.; Ruel, C.; Egelston, C. A.; Guo, W.; Gillece, J. D.; Folkerts, M.; Reining, L. A phase II clinical trial of pembrolizumab and enobosarm in patients with androgen receptor-positive metastatic triple-negative breast cancer. *The Oncologist* **2021**, *26* (2), 99-e217.
(22) Perez, B. A.; Kim, S.; Wang, M.; Karimi, A. M.; Powell, C.; Li, J.; Dilling, T. J.; Chiappori, A.; Latifi, K.; Rose, T. Prospective single-arm phase 1 and 2 study: ipilimumab and nivolumab with thoracic radiation therapy after platinum chemotherapy in extensive-stage small cell lung cancer. *International Journal of Radiation Oncology* Biology* Physics* **2021**, *109* (2), 425-435.
(23) Maity, A.; Mick, R.; Rengan, R.; Mitchell, T. C.; Amaravadi, R. K.; Schuchter, L. M.; Pryma, D. A.; Patsch, D. M.; Maity, A. P.; Minn, A. J. A stratified phase I dose escalation trial of hypofractionated radiotherapy followed by ipilimumab in metastatic melanoma: long-term follow-up and final outcomes. *Oncoimmunology* **2021**, *10* (1), 1863631.
(24) Kitano, S.; Shimizu, T.; Koyama, T.; Ebata, T.; Iwasa, S.; Kondo, S.; Shimomura, A.; Fujiwara, Y.; Yamamoto, N.; Paccaly, A. Dose exploration results from Phase 1 study of cemiplimab, a human monoclonal programmed death (PD)-1 antibody, in Japanese patients with advanced malignancies. *Cancer Chemotherapy and Pharmacology* **2021**, *87*, 53-64.
(25) Mahmood, U.; Bang, A.; Chen, Y.-H.; Mak, R. H.; Lorch, J. H.; Hanna, G. J.; Nishino, M.; Manuszak, C.; Thrash, E. M.; Severgnini, M. A randomized phase 2 study of pembrolizumab with or without radiation in patients with recurrent or metastatic adenoid cystic carcinoma. *International Journal of Radiation Oncology* Biology* Physics* **2021**, *109* (1), 134-144.
(26) Pakkala, S.; Higgins, K.; Chen, Z.; Sica, G.; Steuer, C.; Zhang, C.; Zhang, G.; Wang, S.; Hossain, M. S.; Nazha, B. Durvalumab and tremelimumab with or without stereotactic body radiation therapy in relapsed small cell lung cancer: a randomized phase II study. *Journal for Immunotherapy of Cancer* **2020**, *8* (2).
(27) Majd, N.; Waguespack, S. G.; Janku, F.; Fu, S.; Penas-Prado, M.; Xu, M.; Alshawa, A.; Kamiya-Matsuoka, C.; Raza, S. M.; McCutcheon, I. E. Efficacy of pembrolizumab in patients with pituitary carcinoma: report of four cases from a phase II study. *Journal for ImmunoTherapy of Cancer* **2020**, *8* (2).
(28) van Dijk, N.; Gil-Jimenez, A.; Silina, K.; Hendricksen, K.; Smit, L. A.; de Feijter, J. M.; van Montfoort, M. L.; van Rooijen, C.; Peters, D.; Broeks, A. Preoperative ipilimumab plus nivolumab in locoregionally advanced urothelial cancer: the NABUCCO trial. *Nature medicine* **2020**, *26* (12), 1839-1844.
(29) Gao, J.; Navai, N.; Alhalabi, O.; Siefker-Radtke, A.; Campbell, M. T.; Tidwell, R. S.; Guo, C. C.; Kamat, A. M.; Matin, S. F.; Araujo, J. C. Neoadjuvant PD-L1 plus CTLA-4 blockade in patients with cisplatin-ineligible operable high-risk urothelial carcinoma. *Nature medicine* **2020**, *26* (12), 1845-1851.
(30) Welsh, J. W.; Heymach, J. V.; Guo, C.; Menon, H.; Klein, K.; Cushman, T. R.; Verma, V.; Hess, K. R.; Shroff, G.; Tang, C. Phase 1/2 trial of pembrolizumab and concurrent chemoradiation therapy for limited-stage SCLC. *Journal of Thoracic Oncology* **2020**, *15* (12), 1919-1927.
(31) Duska, L. R.; Scalici, J. M.; Temkin, S. M.; Schwarz, J. K.; Crane, E. K.; Moxley, K. M.; Hamilton, C. A.; Wethington, S. L.; Petroni, G. R.; Varhegyi, N. E. Results of an early safety analysis of a study of the combination of pembrolizumab and pelvic chemoradiation in locally advanced cervical cancer. *Cancer* **2020**, *126* (22), 4948-4956.

(32) Schweizer, C.; Schubert, P.; Rutzner, S.; Eckstein, M.; Haderlein, M.; Lettmaier, S.; Semrau, S.; Gostian, A.-O.; Frey, B.; Gaipl, U. S. Prospective evaluation of the prognostic value of immune-related adverse events in patients with non-melanoma solid tumour treated with PD-1/PD-L1 inhibitors alone and in combination with radiotherapy. *European Journal of Cancer* **2020**, *140*, 55-62.
(33) Takahashi, A.; Namikawa, K.; Ogata, D.; Nakano, E.; Jinnai, S.; Nakama, K.; Tsutsui, K.; Muto, Y.; Mizuta, H.; Yamazaki, N. Real-world efficacy and safety data of nivolumab and ipilimumab combination therapy in Japanese patients with advanced melanoma. *The Journal of Dermatology* **2020**, *47* (11), 1267-1275.
(34) Awada, G.; Salama, L. B.; De Cremer, J.; Schwarze, J. K.; Fischbuch, L.; Seynaeve, L.; Du Four, S.; Vanbinst, A.-M.; Michotte, A.; Everaert, H. Axitinib plus avelumab in the treatment of recurrent glioblastoma: a stratified, open-label, single-center phase 2 clinical trial (GliAvAx). *Journal for immunotherapy of cancer* **2020**, *8* (2).
(35) Welsh, J.; Menon, H.; Chen, D.; Verma, V.; Tang, C.; Altan, M.; Hess, K.; De Groot, P.; Nguyen, Q.-N.; Varghese, R. Pembrolizumab with or without radiation therapy for metastatic non-small cell lung cancer: a randomized phase I/II trial. *Journal for immunotherapy of cancer* **2020**, *8* (2).
(36) Wang, Z.; Ying, J.; Xu, J.; Yuan, P.; Duan, J.; Bai, H.; Guo, C.; Li, L.; Yang, Z.; Wan, R. Safety, Antitumor Activity, and Pharmacokinetics of Toripalimab, a Programmed Cell Death 1 Inhibitor, in Patients With Advanced Non–Small Cell Lung Cancer: A Phase 1 Trial. *JAMA network open* **2020**, *3* (10), e2013770-e2013770.
(37) Schoenfeld, J. D.; Hanna, G. J.; Jo, V. Y.; Rawal, B.; Chen, Y.-H.; Catalano, P. S.; Lako, A.; Ciantra, Z.; Weirather, J. L.; Criscitiello, S. Neoadjuvant nivolumab or nivolumab plus ipilimumab in untreated oral cavity squamous cell carcinoma: a phase 2 open-label randomized clinical trial. *JAMA oncology* **2020**, *6* (10), 1563-1570.
(38) O'Neill, C.; Hayat, T.; Hamm, J.; Healey, M.; Zheng, Q.; Li, Y.; Martin II, R. C. A phase 1b trial of concurrent immunotherapy and irreversible electroporation in the treatment of locally advanced pancreatic adenocarcinoma. *Surgery* **2020**, *168* (4), 610-616.
(39) Frumovitz, M.; Westin, S. N.; Salvo, G.; Zarifa, A.; Xu, M.; Yap, T. A.; Rodon, A. J.; Karp, D. D.; Abonofal, A.; Jazaeri, A. A. Phase II study of pembrolizumab efficacy and safety in women with recurrent small cell neuroendocrine carcinoma of the lower genital tract. *Gynecologic oncology* **2020**, *158* (3), 570-575.
(40) Ratnayake, G.; Reinwald, S.; Shackleton, M.; Moore, M.; Voskoboynik, M.; Ruben, J.; van Zelm, M. C.; Yu, D.; Ward, R.; Smith, R. Stereotactic radiation therapy combined with immunotherapy against metastatic melanoma: long-term results of a phase 1 clinical trial. *International Journal of Radiation Oncology* Biology* Physics* **2020**, *108* (1), 150-156.
(41) Boutros, C.; Chaput-Gras, N.; Lanoy, E.; Larive, A.; Mateus, C.; Routier, E.; Sun, R.; Tao, Y. G.; Massard, C.; Bahleda, R. Dose escalation phase 1 study of radiotherapy in combination with anti-cytotoxic-T-lymphocyte-associated antigen 4 monoclonal antibody ipilimumab in patients with metastatic melanoma. *Journal for Immunotherapy of Cancer* **2020**, *8* (2).
(42) Kawazoe, A.; Fukuoka, S.; Nakamura, Y.; Kuboki, Y.; Wakabayashi, M.; Nomura, S.; Mikamoto, Y.; Shima, H.; Fujishiro, N.; Higuchi, T. Lenvatinib plus pembrolizumab in patients with advanced gastric cancer in the first-line or second-line setting (EPOC1706): an open-label, single-arm, phase 2 trial. *The Lancet Oncology* **2020**, *21* (8), 1057-1065.
(43) Wei, X. L.; Ren, C.; Wang, F. H.; Zhang, Y.; Zhao, H. Y.; Zou, B. Y.; Wang, Z. Q.; Qiu, M. Z.; Zhang, D. S.; Luo, H. Y. A phase I study of toripalimab, an anti-PD-1 antibody, in patients with refractory malignant solid tumors. *Cancer Communications* **2020**, *40* (8), 345-354.
(44) Brastianos, P. K.; Lee, E. Q.; Cohen, J. V.; Tolaney, S. M.; Lin, N. U.; Wang, N.; Chukwueke, U.; White, M. D.; Nayyar, N.; Kim, A. Single-arm, open-label phase 2 trial of pembrolizumab in patients with leptomeningeal carcinomatosis. *Nature medicine* **2020**, *26* (8), 1280-1284.
(45) Kim, C.; Liu, S. V.; Subramaniam, D. S.; Torres, T.; Loda, M.; Esposito, G.; Giaccone, G. Phase I study of the 177Lu-DOTA0-Tyr3-Octreotate (lutathera) in combination with nivolumab in patients with neuroendocrine tumors of the lung. *Journal for Immunotherapy of Cancer* **2020**, *8* (2).
(46) Graff, J. N.; Beer, T. M.; Alumkal, J. J.; Slottke, R. E.; Redmond, W. L.; Thomas, G. V.; Thompson, R. F.; Wood, M. A.; Koguchi, Y.; Chen, Y. A phase II single-arm study of pembrolizumab with enzalutamide in men with metastatic castration-resistant prostate cancer progressing on enzalutamide alone. *Journal for immunotherapy of cancer* **2020**, *8* (2).
(47) Ferrarotto, R.; Bell, D.; Rubin, M. L.; Hutcheson, K. A.; Johnson, J. M.; Goepfert, R. P.; Phan, J.; Elamin, Y. Y.; Torman, D. K.; Warneke, C. L. Impact of neoadjuvant durvalumab with or without tremelimumab on CD8+ tumor lymphocyte density, safety, and efficacy in patients with oropharynx cancer: CIAO trial results. *Clinical Cancer Research* **2020**, *26* (13), 3211-3219.

(48) Postow, M. A.; Knox, S. J.; Goldman, D. A.; Elhanati, Y.; Mavinkurve, V.; Wong, P.; Halpenny, D.; Reddy, S. K.; Vado, K.; McCabe, D. A prospective, phase 1 trial of nivolumab, ipilimumab, and radiotherapy in patients with advanced melanoma. *Clinical Cancer Research* **2020**, *26* (13), 3193-3201.
(49) Zamarin, D.; Walderich, S.; Holland, A.; Zhou, Q.; Iasonos, A. E.; Torrisi, J. M.; Merghoub, T.; Chesebrough, L. F.; Mcdonnell, A. S.; Gallagher, J. M. Safety, immunogenicity, and clinical efficacy of durvalumab in combination with folate receptor alpha vaccine TPIV200 in patients with advanced ovarian cancer: a phase II trial. *Journal for ImmunoTherapy of Cancer* **2020**, *8* (1).
(50) Feng, K.; Liu, Y.; Zhao, Y.; Yang, Q.; Dong, L.; Liu, J.; Li, X.; Zhao, Z.; Mei, Q.; Han, W. Efficacy and biomarker analysis of nivolumab plus gemcitabine and cisplatin in patients with unresectable or metastatic biliary tract cancers: results from a phase II study. *Journal for immunotherapy of cancer* **2020**, *8* (1).
(51) Kanda, S.; Ohe, Y.; Goto, Y.; Horinouchi, H.; Fujiwara, Y.; Nokihara, H.; Yamamoto, N.; Yamamoto, T.; Tamura, T. Five-year safety and efficacy data from a phase Ib study of nivolumab and chemotherapy in advanced non-small-cell lung cancer. *Cancer Science* **2020**, *111* (6), 1933-1942.
(52) Arrieta, O.; Barrón, F.; Ramírez-Tirado, L. A.; Zatarain-Barrón, Z. L.; Cardona, A. F.; Díaz-García, D.; Ramos, M. Y.; Mota-Vega, B.; Carmona, A.; Álvarez, M. P. P. Efficacy and safety of pembrolizumab plus docetaxel vs docetaxel alone in patients with previously treated advanced non–small cell lung cancer: The PROLUNG phase 2 randomized clinical trial. *JAMA oncology* **2020**, *6* (6), 856-864.
(53) Rozeman, E. A.; Prevoo, W.; Meier, M. A.; Sikorska, K.; Van, T. M.; van de Wiel, B. A.; van Der Wal, J. E.; Mallo, H. A.; Grijpink-Ongering, L. G.; Broeks, A. Phase Ib/II trial testing combined radiofrequency ablation and ipilimumab in uveal melanoma (SECIRA-UM). *Melanoma Research* **2020**, *30* (3), 252-260.
(54) Xie, C.; Duffy, A. G.; Brar, G.; Fioravanti, S.; Mabry-Hrones, D.; Walker, M.; Bonilla, C. M.; Wood, B. J.; Citrin, D. E.; Gil Ramirez, E. M. Immune checkpoint blockade in combination with stereotactic body radiotherapy in patients with metastatic pancreatic ductal adenocarcinoma. *Clinical Cancer Research* **2020**, *26* (10), 2318-2326.
(55) Music, M.; Iafolla, M.; Soosaipillai, A.; Batruch, I.; Prassas, I.; Pintilie, M.; Hansen, A. R.; Bedard, P. L.; Lheureux, S.; Spreafico, A. Predicting response and toxicity to PD-1 inhibition using serum autoantibodies identified from immuno-mass spectrometry. *F1000Research* **2020**, *9*.
(56) Liu, J.; Liu, Q.; Li, Y.; Li, Q.; Su, F.; Yao, H.; Su, S.; Wang, Q.; Jin, L.; Wang, Y. Efficacy and safety of camrelizumab combined with apatinib in advanced triple-negative breast cancer: an open-label phase II trial. *Journal for immunotherapy of cancer* **2020**, *8* (1).
(57) Xie, L.; Xu, J.; Sun, X.; Guo, W.; Gu, J.; Liu, K.; Zheng, B.; Ren, T.; Huang, Y.; Tang, X. Apatinib plus camrelizumab (anti-PD1 therapy, SHR-1210) for advanced osteosarcoma (APFAO) progressing after chemotherapy: a single-arm, open-label, phase 2 trial. *Journal for immunotherapy of cancer* **2020**, *8* (1).
(58) Goldberg, S. B.; Schalper, K. A.; Gettinger, S. N.; Mahajan, A.; Herbst, R. S.; Chiang, A. C.; Lilenbaum, R.; Wilson, F. H.; Omay, S. B.; James, B. Y. Pembrolizumab for management of patients with NSCLC and brain metastases: long-term results and biomarker analysis from a non-randomised, open-label, phase 2 trial. *The Lancet Oncology* **2020**, *21* (5), 655-663.
(59) Gao, S.; Li, N.; Gao, S.; Xue, Q.; Ying, J.; Wang, S.; Tao, X.; Zhao, J.; Mao, Y.; Wang, B. Neoadjuvant PD-1 inhibitor (Sintilimab) in NSCLC. *Journal of Thoracic Oncology* **2020**, *15* (5), 816-826.
(60) Kamath, S. D.; Kalyan, A.; Kircher, S.; Nimeiri, H.; Fought, A. J.; Benson III, A.; Mulcahy, M. Ipilimumab and gemcitabine for advanced pancreatic cancer: a phase Ib study. *The oncologist* **2020**, *25* (5), e808-e815.
(61) Naing, A.; Meric-Bernstam, F.; Stephen, B.; Karp, D. D.; Hajjar, J.; Ahnert, J. R.; Piha-Paul, S. A.; Colen, R. R.; Jimenez, C.; Raghav, K. P. Phase 2 study of pembrolizumab in patients with advanced rare cancers. *Journal for immunotherapy of cancer* **2020**, *8* (1).
(62) Kelly, C. M.; Antonescu, C. R.; Bowler, T.; Munhoz, R.; Chi, P.; Dickson, M. A.; Gounder, M. M.; Keohan, M. L.; Movva, S.; Dholakia, R. Objective response rate among patients with locally advanced or metastatic sarcoma treated with talimogene laherparepvec in combination with pembrolizumab: a phase 2 clinical trial. *JAMA oncology* **2020**, *6* (3), 402-408.
(63) Ross, A. E.; Hurley, P. J.; Tran, P. T.; Rowe, S. P.; Benzon, B.; Neal, T. O.; Chapman, C.; Harb, R.; Milman, Y.; Trock, B. J. A pilot trial of pembrolizumab plus prostatic cryotherapy for men with newly diagnosed oligometastatic hormone-sensitive prostate cancer. *Prostate cancer and prostatic diseases* **2020**, *23* (1), 184-193.

(64) Rodriguez, C. P.; Wu, Q.; Voutsinas, J.; Fromm, J. R.; Jiang, X.; Pillarisetty, V. G.; Lee, S. M.; Santana-Davila, R.; Goulart, B.; Baik, C. S. A phase II trial of pembrolizumab and vorinostat in recurrent metastatic head and neck squamous cell carcinomas and salivary gland cancer. *Clinical Cancer Research* **2020**, *26* (4), 837-845.
(65) Shah, A. N.; Flaum, L.; Helenowski, I.; Santa-Maria, C. A.; Jain, S.; Rademaker, A.; Nelson, V.; Tsarwhas, D.; Cristofanilli, M.; Gradishar, W. Phase II study of pembrolizumab and capecitabine for triple negative and hormone receptor-positive, HER2− negative endocrine-refractory metastatic breast cancer. *Journal for immunotherapy of cancer* **2020**, *8* (1).
(66) Yarchoan, M.; Huang, C. Y.; Zhu, Q.; Ferguson, A. K.; Durham, J. N.; Anders, R. A.; Thompson, E. D.; Rozich, N. S.; Thomas, D. L.; Nauroth, J. M. A phase 2 study of GVAX colon vaccine with cyclophosphamide and pembrolizumab in patients with mismatch repair proficient advanced colorectal cancer. *Cancer medicine* **2020**, *9* (4), 1485-1494.
(67) Lin, S. H.; Lin, Y.; Yao, L.; Kalhor, N.; Carter, B. W.; Altan, M.; Blumenschein, G.; Byers, L. A.; Fossella, F.; Gibbons, D. L. Phase II trial of concurrent atezolizumab with chemoradiation for unresectable NSCLC. *Journal of Thoracic Oncology* **2020**, *15* (2), 248-257.
(68) Welsh, J. W.; Heymach, J. V.; Chen, D.; Verma, V.; Cushman, T. R.; Hess, K. R.; Shroff, G.; Tang, C.; Skoulidis, F.; Jeter, M. Phase I trial of pembrolizumab and radiation therapy after induction chemotherapy for extensive-stage small cell lung cancer. *Journal of Thoracic Oncology* **2020**, *15* (2), 266-273.
(69) Mahalingam, D.; Wilkinson, G. A.; Eng, K. H.; Fields, P.; Raber, P.; Moseley, J. L.; Cheetham, K.; Coffey, M.; Nuovo, G.; Kalinski, P. Pembrolizumab in combination with the oncolytic virus pelareorep and chemotherapy in patients with advanced pancreatic adenocarcinoma: a phase Ib study. *Clinical Cancer Research* **2020**, *26* (1), 71-81.
(70) Elbers, J. B.; Al-Mamgani, A.; Tesseslaar, M. E.; van den Brekel, M. W.; Lange, C. A.; van der Wal, J. E.; Verheij, M.; Zuur, C. L.; de Boer, J. P. Immuno-radiotherapy with cetuximab and avelumab for advanced stage head and neck squamous cell carcinoma: Results from a phase-I trial. *Radiotherapy and Oncology* **2020**, *142*, 79-84.
(71) Di Giacomo, A. M.; Covre, A.; Finotello, F.; Rieder, D.; Danielli, R.; Sigalotti, L.; Giannarelli, D.; Petitprez, F.; Lacroix, L.; Valente, M. Guadecitabine plus ipilimumab in unresectable melanoma: the NIBIT-M4 clinical trial. *Clinical Cancer Research* **2019**, *25* (24), 7351-7362.
(72) Welsh, J. W.; Tang, C.; De Groot, P.; Naing, A.; Hess, K. R.; Heymach, J. V.; Papadimitrakopoulou, V. A.; Cushman, T. R.; Subbiah, V.; Chang, J. Y. Phase II trial of ipilimumab with stereotactic radiation therapy for metastatic disease: outcomes, toxicities, and low-dose radiation–related abscopal responses. *Cancer immunology research* **2019**, *7* (12), 1903-1909.
(73) Gray, J. E.; Saltos, A.; Tanvetyanon, T.; Haura, E. B.; Creelan, B.; Antonia, S. J.; Shafique, M.; Zheng, H.; Dai, W.; Saller, J. J. Phase I/Ib study of pembrolizumab plus vorinostat in advanced/metastatic non–small cell lung cancer. *Clinical Cancer Research* **2019**, *25* (22), 6623-6632.
(74) Qiao, G.; Wang, X.; Zhou, X.; Morse, M. A.; Wu, J.; Wang, S.; Song, Y.; Jiang, N.; Zhao, Y.; Zhou, L. Immune correlates of clinical benefit in a phase I study of hyperthermia with adoptive T cell immunotherapy in patients with solid tumors. *International Journal of Hyperthermia* **2019**, *36* (sup1), 74-82.
(75) Chintakuntlawar, A. V.; Yin, J.; Foote, R. L.; Kasperbauer, J. L.; Rivera, M.; Asmus, E.; Garces, N. I.; Janus, J. R.; Liu, M.; Ma, D. J. A phase 2 study of pembrolizumab combined with chemoradiotherapy as initial treatment for anaplastic thyroid cancer. *Thyroid* **2019**, *29* (11), 1615-1622.
(76) Feun, L. G.; Li, Y. Y.; Wu, C.; Wangpaichitr, M.; Jones, P. D.; Richman, S. P.; Madrazo, B.; Kwon, D.; Garcia-Buitrago, M.; Martin, P. Phase 2 study of pembrolizumab and circulating biomarkers to predict anticancer response in advanced, unresectable hepatocellular carcinoma. *Cancer* **2019**, *125* (20), 3603-3614.
(77) Habra, M. A.; Stephen, B.; Campbell, M.; Hess, K.; Tapia, C.; Xu, M.; Rodon Ahnert, J.; Jimenez, C.; Lee, J. E.; Perrier, N. D. Phase II clinical trial of pembrolizumab efficacy and safety in advanced adrenocortical carcinoma. *Journal for immunotherapy of cancer* **2019**, *7*, 1-9.
(78) Cho, J.; Kim, H. S.; Ku, B. M.; Choi, Y.-L.; Cristescu, R.; Han, J.; Sun, J.-M.; Lee, S.-H.; Ahn, J. S.; Park, K. Pembrolizumab for patients with refractory or relapsed thymic epithelial tumor: an open-label phase II trial. *J clin oncol* **2019**, *37* (24), 2162-2170.
(79) Chalmers, A. W.; Patel, S.; Boucher, K.; Cannon, L.; Esplin, M.; Luckart, J.; Graves, N.; Van Duren, T.; Akerley, W. Phase I trial of targeted EGFR or ALK therapy with ipilimumab in metastatic NSCLC with long-term follow-up. *Targeted Oncology* **2019**, *14*, 417-421.

(80) Mego, M.; Svetlovska, D.; Chovanec, M.; Rečkova, M.; Rejlekova, K.; Obertova, J.; Palacka, P.; Sycova-Mila, Z.; De Giorgi, U.; Mardiak, J. Phase II study of avelumab in multiple relapsed/refractory germ cell cancer. *Investigational new drugs* **2019**, *37*, 748-754.
(81) Thomas, A.; Vilimas, R.; Trindade, C.; Erwin-Cohen, R.; Roper, N.; Xi, L.; Krishnasamy, V.; Levy, E.; Mammen, A.; Nichols, S. Durvalumab in combination with olaparib in patients with relapsed SCLC: results from a phase II study. *Journal of Thoracic Oncology* **2019**, *14* (8), 1447-1457.
(82) Zimmer, A. S.; Nichols, E.; Cimino-Mathews, A.; Peer, C.; Cao, L.; Lee, M.-J.; Kohn, E. C.; Annunziata, C. M.; Lipkowitz, S.; Trepel, J. B. A phase I study of the PD-L1 inhibitor, durvalumab, in combination with a PARP inhibitor, olaparib, and a VEGFR1–3 inhibitor, cediranib, in recurrent women's cancers with biomarker analyses. *Journal for immunotherapy of cancer* **2019**, *7* (1), 1-8.
(83) Suresh, K.; Naidoo, J.; Zhong, Q.; Xiong, Y.; Mammen, J.; De Flores, M. V.; Cappelli, L.; Balaji, A.; Palmer, T.; Forde, P. M. The alveolar immune cell landscape is dysregulated in checkpoint inhibitor pneumonitis. *The Journal of clinical investigation* **2019**, *129* (10), 4305-4315.
(84) Sundahl, N.; Seremet, T.; Van Dorpe, J.; Neyns, B.; Ferdinande, L.; Meireson, A.; Brochez, L.; Kruse, V.; Ost, P. Phase 2 trial of nivolumab combined with stereotactic body radiation therapy in patients with metastatic or locally advanced inoperable melanoma. *International Journal of Radiation Oncology* Biology* Physics* **2019**, *104* (4), 828-835.
(85) Voorwerk, L.; Slagter, M.; Horlings, H. M.; Sikorska, K.; van de Vijver, K. K.; de Maaker, M.; Nederlof, I.; Kluin, R. J.; Warren, S.; Ong, S. Immune induction strategies in metastatic triple-negative breast cancer to enhance the sensitivity to PD-1 blockade: the TONIC trial. *Nature medicine* **2019**, *25* (6), 920-928.
(86) Wilky, B. A.; Trucco, M. M.; Subhawong, T. K.; Florou, V.; Park, W.; Kwon, D.; Wieder, E. D.; Kolonias, D.; Rosenberg, A. E.; Kerr, D. A. Axitinib plus pembrolizumab in patients with advanced sarcomas including alveolar soft-part sarcoma: a single-centre, single-arm, phase 2 trial. *The lancet oncology* **2019**, *20* (6), 837-848.
(87) Sundahl, N.; Vandekerkhove, G.; Decaestecker, K.; Meireson, A.; De Visschere, P.; Fonteyne, V.; De Maeseneer, D.; Reynders, D.; Goetghebeur, E.; Van Dorpe, J. Randomized phase 1 trial of pembrolizumab with sequential versus concomitant stereotactic body radiotherapy in metastatic urothelial carcinoma. *European urology* **2019**, *75* (5), 707-711.
(88) Disselhorst, M. J.; Quispel-Janssen, J.; Lalezari, F.; Monkhorst, K.; de Vries, J. F.; van der Noort, V.; Harms, E.; Burgers, S.; Baas, P. Ipilimumab and nivolumab in the treatment of recurrent malignant pleural mesothelioma (INITIATE): results of a prospective, single-arm, phase 2 trial. *The Lancet Respiratory Medicine* **2019**, *7* (3), 260-270.
(89) Huang, J.; Mo, H.; Zhang, W.; Chen, X.; Qu, D.; Wang, X.; Wu, D.; Wang, X.; Lan, B.; Yang, B. Promising efficacy of SHR-1210, a novel anti–programmed cell death 1 antibody, in patients with advanced gastric and gastroesophageal junction cancer in China. *Cancer* **2019**, *125* (5), 742-749.
(90) Chung, V.; Kos, F.; Hardwick, N.; Yuan, Y.; Chao, J.; Li, D.; Waisman, J.; Li, M.; Zurcher, K.; Frankel, P. Evaluation of safety and efficacy of p53MVA vaccine combined with pembrolizumab in patients with advanced solid cancers. *Clinical and Translational Oncology* **2019**, *21*, 363-372.
(91) Miyamoto, S.; Nomura, R.; Sato, K.; Awano, N.; Kuse, N.; Inomata, M.; Izumo, T.; Terada, Y.; Furuhata, Y.; Bae, Y. Nivolumab and stereotactic radiation therapy for the treatment of patients with Stage IV non-small-cell lung cancer. *Japanese journal of clinical oncology* **2019**, *49* (2), 160-164.
(92) Tang, B.; Yan, X.; Sheng, X.; Si, L.; Cui, C.; Kong, Y.; Mao, L.; Lian, B.; Bai, X.; Wang, X. Safety and clinical activity with an anti-PD-1 antibody JS001 in advanced melanoma or urologic cancer patients. *Journal of hematology & oncology* **2019**, *12*, 1-15.
(93) Ali, O. H.; Berner, F.; Bomze, D.; Fässler, M.; Diem, S.; Cozzio, A.; Jörger, M.; Früh, M.; Driessen, C.; Lenz, T. L. Human leukocyte antigen variation is associated with adverse events of checkpoint inhibitors. *European Journal of Cancer* **2019**, *107*, 8-14.
(94) Kluger, H. M.; Chiang, V.; Mahajan, A.; Zito, C. R.; Sznol, M.; Tran, T.; Weiss, S. A.; Cohen, J. V.; Yu, J.; Hegde, U. Long-term survival of patients with melanoma with active brain metastases treated with pembrolizumab on a phase II trial. *Journal of Clinical Oncology* **2019**, *37* (1), 52.
(95) Massarelli, E.; William, W.; Johnson, F.; Kies, M.; Ferrarotto, R.; Guo, M.; Feng, L.; Lee, J. J.; Tran, H.; Kim, Y. U. Combining immune checkpoint blockade and tumor-specific vaccine for patients with incurable human papillomavirus 16–related cancer: a phase 2 clinical trial. *JAMA oncology* **2019**, *5* (1), 67-73.

(96) Parikh, M.; Pan, C.-X.; Beckett, L. A.; Li, Y.; Robles, D. A.; Aujla, P. K.; Lara Jr, P. N. Pembrolizumab combined with either docetaxel or gemcitabine in patients with advanced or metastatic platinum-refractory urothelial cancer: results from a phase I study. *Clinical genitourinary cancer* **2018**, *16* (6), 421-428. e421.
(97) Maity, A.; Mick, R.; Huang, A. C.; George, S. M.; Farwell, M. D.; Lukens, J. N.; Berman, A. T.; Mitchell, T. C.; Bauml, J.; Schuchter, L. M. A phase I trial of pembrolizumab with hypofractionated radiotherapy in patients with metastatic solid tumours. *British journal of cancer* **2018**, *119* (10), 1200-1207.
(98) Amaria, R. N.; Reddy, S. M.; Tawbi, H. A.; Davies, M. A.; Ross, M. I.; Glitza, I. C.; Cormier, J. N.; Lewis, C.; Hwu, W.-J.; Hanna, E. Neoadjuvant immune checkpoint blockade in high-risk resectable melanoma. *Nature medicine* **2018**, *24* (11), 1649-1654.
(99) Tobin, R. P.; Jordan, K. R.; Robinson, W. A.; Davis, D.; Borges, V. F.; Gonzalez, R.; Lewis, K. D.; McCarter, M. D. Targeting myeloid-derived suppressor cells using all-trans retinoic acid in melanoma patients treated with Ipilimumab. *International immunopharmacology* **2018**, *63*, 282-291.
(100) Tree, A. C.; Jones, K.; Hafeez, S.; Sharabiani, M. T. A.; Harrington, K. J.; Lalondrelle, S.; Ahmed, M.; Huddart, R. A. Dose-limiting urinary toxicity with pembrolizumab combined with weekly hypofractionated radiation therapy in bladder cancer. *International Journal of Radiation Oncology* Biology* Physics* **2018**, *101* (5), 1168-1171.
(101) Lisberg, A.; Cummings, A.; Goldman, J. W.; Bornazyan, K.; Reese, N.; Wang, T.; Coluzzi, P.; Ledezma, B.; Mendenhall, M.; Hunt, J. A phase II study of pembrolizumab in EGFR-mutant, PD-L1+, tyrosine kinase inhibitor naïve patients with advanced NSCLC. *Journal of Thoracic Oncology* **2018**, *13* (8), 1138-1145.
(102) Calabrò, L.; Morra, A.; Giannarelli, D.; Amato, G.; D'Incecco, A.; Covre, A.; Lewis, A.; Rebelatto, M. C.; Danielli, R.; Altomonte, M. Tremelimumab combined with durvalumab in patients with mesothelioma (NIBIT-MESO-1): an open-label, non-randomised, phase 2 study. *The Lancet Respiratory Medicine* **2018**, *6* (6), 451-460.
(103) Luke, J. J.; Lemons, J. M.; Karrison, T. G.; Pitroda, S. P.; Melotek, J. M.; Zha, Y.; Al-Hallaq, H. A.; Arina, A.; Khodarev, N. N.; Janisch, L. Safety and clinical activity of pembrolizumab and multisite stereotactic body radiotherapy in patients with advanced solid tumors. *Journal of Clinical Oncology* **2018**, *36* (16), 1611.
(104) Strauss, J.; Heery, C. R.; Schlom, J.; Madan, R. A.; Cao, L.; Kang, Z.; Lamping, E.; Marté, J. L.; Donahue, R. N.; Grenga, I. Phase I trial of M7824 (MSB0011359C), a bifunctional fusion protein targeting PD-L1 and TGFβ, in advanced solid tumors. *Clinical Cancer Research* **2018**, *24* (6), 1287-1295.
(105) Da Gama Duarte, J.; Parakh, S.; Andrews, M. C.; Woods, K.; Pasam, A.; Tutuka, C.; Ostrouska, S.; Blackburn, J. M.; Behren, A.; Cebon, J. Autoantibodies may predict immune-related toxicity: results from a Phase I study of intralesional Bacillus Calmette–Guérin followed by ipilimumab in patients with advanced metastatic melanoma. *Frontiers in immunology* **2018**, *9*, 411.
(106) Giaccone, G.; Kim, C.; Thompson, J.; McGuire, C.; Kallakury, B.; Chahine, J. J.; Manning, M.; Mogg, R.; Blumenschein, W. M.; Tan, M. T. Pembrolizumab in patients with thymic carcinoma: a single-arm, single-centre, phase 2 study. *The Lancet Oncology* **2018**, *19* (3), 347-355.
(107) Yang, C.-F. J.; McSherry, F.; Mayne, N. R.; Wang, X.; Berry, M. F.; Tong, B.; Harpole Jr, D. H.; D'Amico, T. A.; Christensen, J. D.; Ready, N. E. Surgical outcomes after neoadjuvant chemotherapy and ipilimumab for non-small cell lung cancer. *The Annals of Thoracic Surgery* **2018**, *105* (3), 924-929.
(108) Sakamuri, D.; Glitza, I. C.; Betancourt Cuellar, S. L.; Subbiah, V.; Fu, S.; Tsimberidou, A. M.; Wheler, J. J.; Hong, D. S.; Naing, A.; Falchook, G. S. Phase I dose-escalation study of anti–CTLA-4 antibody ipilimumab and lenalidomide in patients with advanced cancers. *Molecular cancer therapeutics* **2018**, *17* (3), 671-676.
(109) Ariyan, C. E.; Brady, M. S.; Siegelbaum, R. H.; Hu, J.; Bello, D. M.; Rand, J.; Fisher, C.; Lefkowitz, R. A.; Panageas, K. S.; Pulitzer, M. Robust antitumor responses result from local chemotherapy and CTLA-4 blockade. *Cancer immunology research* **2018**, *6* (2), 189-200.
(110) Haag, G.; Zoernig, I.; Hassel, J.; Halama, N.; Dick, J.; Lang, N.; Podola, L.; Funk, J.; Ziegelmeier, C.; Juenger, S. Phase II trial of ipilimumab in melanoma patients with preexisting humoural immune response to NY-ESO-1. *European Journal of Cancer* **2018**, *90*, 122-129.
(111) Weiss, G. J.; Blaydorn, L.; Beck, J.; Bornemann-Kolatzki, K.; Urnovitz, H.; Schütz, E.; Khemka, V. Phase Ib/II study of gemcitabine, nab-paclitaxel, and pembrolizumab in metastatic pancreatic adenocarcinoma. *Investigational new drugs* **2018**, *36*, 96-102.

(112) Shoushtari, A. N.; Friedman, C. F.; Navid-Azarbaijani, P.; Postow, M. A.; Callahan, M. K.; Momtaz, P.; Panageas, K. S.; Wolchok, J. D.; Chapman, P. B. Measuring toxic effects and time to treatment failure for nivolumab plus ipilimumab in melanoma. *JAMA oncology* **2018**, *4* (1), 98-101.
(113) Patel, S. P.; Kim, D. W.; Bassett, R. L.; Cain, S.; Washington, E.; Hwu, W.-J.; Kim, K. B.; Papadopoulos, N. E.; Homsi, J.; Hwu, P. A phase II study of ipilimumab plus temozolomide in patients with metastatic melanoma. *Cancer Immunology, Immunotherapy* **2017**, *66*, 1359-1366.
(114) Ben-Ami, E.; Barysauskas, C. M.; Solomon, S.; Tahlil, K.; Malley, R.; Hohos, M.; Polson, K.; Loucks, M.; Severgnini, M.; Patel, T. Immunotherapy with single agent nivolumab for advanced leiomyosarcoma of the uterus: results of a phase 2 study. *Cancer* **2017**, *123* (17), 3285-3290.
(115) Qin, A.; Street, L.; Cease, K.; Viglianti, B. L.; Warren, E. H.; Zhao, L.; Ramnath, N. Clinical determinants of durable clinical benefit of pembrolizumab in veterans with advanced non–small-cell lung cancer. *Clinical lung cancer* **2017**, *18* (5), 559-564.
(116) Weiss, G. J.; Waypa, J.; Blaydorn, L.; Coats, J.; McGahey, K.; Sangal, A.; Niu, J.; Lynch, C. A.; Farley, J. H.; Khemka, V. A phase Ib study of pembrolizumab plus chemotherapy in patients with advanced cancer (PembroPlus). *British journal of cancer* **2017**, *117* (1), 33-40.
(117) D'Angelo, S. P.; Shoushtari, A. N.; Keohan, M. L.; Dickson, M. A.; Gounder, M. M.; Chi, P.; Loo, J. K.; Gaffney, L.; Schneider, L.; Patel, Z. Combined KIT and CTLA-4 blockade in patients with refractory GIST and other advanced sarcomas: a phase Ib study of dasatinib plus ipilimumab. *Clinical Cancer Research* **2017**, *23* (12), 2972-2980.
(118) Reilley, M. J.; Bailey, A.; Subbiah, V.; Janku, F.; Naing, A.; Falchook, G.; Karp, D.; Piha-Paul, S.; Tsimberidou, A.; Fu, S. Phase I clinical trial of combination imatinib and ipilimumab in patients with advanced malignancies. *Journal for immunotherapy of cancer* **2017**, *5*, 1-10.
(119) Weide, B.; Martens, A.; Wistuba-Hamprecht, K.; Zelba, H.; Maier, L.; Lipp, H.-P.; Klumpp, B. D.; Soffel, D.; Eigentler, T. K.; Garbe, C. Combined treatment with ipilimumab and intratumoral interleukin-2 in pretreated patients with stage IV melanoma—safety and efficacy in a phase II study. *Cancer Immunology, Immunotherapy* **2017**, *66*, 441-449.
(120) Yamamoto, N.; Nokihara, H.; Yamada, Y.; Shibata, T.; Tamura, Y.; Seki, Y.; Honda, K.; Tanabe, Y.; Wakui, H.; Tamura, T. Phase I study of Nivolumab, an anti-PD-1 antibody, in patients with malignant solid tumors. *Investigational new drugs* **2017**, *35*, 207-216.
(121) Brustugun, O.; Sprauten, M.; Helland, Å. Real-world data on nivolumab treatment of non-small cell lung cancer. *Acta Oncologica* **2017**, *56* (3), 438-440.
(122) Duffy, A. G.; Ulahannan, S. V.; Makorova-Rusher, O.; Rahma, O.; Wedemeyer, H.; Pratt, D.; Davis, J. L.; Hughes, M. S.; Heller, T.; ElGindi, M. Tremelimumab in combination with ablation in patients with advanced hepatocellular carcinoma. *Journal of hepatology* **2017**, *66* (3), 545-551.
(123) Kanda, S.; Goto, K.; Shiraishi, H.; Kubo, E.; Tanaka, A.; Utsumi, H.; Sunami, K.; Kitazono, S.; Mizugaki, H.; Horinouchi, H. Safety and efficacy of nivolumab and standard chemotherapy drug combination in patients with advanced non-small-cell lung cancer: a four arms phase Ib study. *Annals of Oncology* **2016**, *27* (12), 2242-2250.
(124) Ray, A.; Williams, M. A.; Meek, S. M.; Bowen, R. C.; Grossmann, K. F.; Andtbacka, R. H.; Bowles, T. L.; Hyngstrom, J. R.; Leachman, S. A.; Grossman, D. A phase I study of intratumoral ipilimumab and interleukin-2 in patients with advanced melanoma. *Oncotarget* **2016**, *7* (39), 64390.
(125) Graff, J. N.; Alumkal, J. J.; Drake, C. G.; Thomas, G. V.; Redmond, W. L.; Farhad, M.; Cetnar, J. P.; Ey, F. S.; Bergan, R. C.; Slottke, R. Early evidence of anti-PD-1 activity in enzalutamide-resistant prostate cancer. *Oncotarget* **2016**, *7* (33), 52810.
(126) Bjoern, J.; Iversen, T. Z.; Nitschke, N. J.; Andersen, M. H.; Svane, I. M. Safety, immune and clinical responses in metastatic melanoma patients vaccinated with a long peptide derived from indoleamine 2, 3-dioxygenase in combination with ipilimumab. *Cytotherapy* **2016**, *18* (8), 1043-1055.
(127) Goldberg, S. B.; Gettinger, S. N.; Mahajan, A.; Chiang, A. C.; Herbst, R. S.; Sznol, M.; Tsiouris, A. J.; Cohen, J.; Vortmeyer, A.; Jilaveanu, L. Pembrolizumab for patients with melanoma or non-small-cell lung cancer and untreated brain metastases: early analysis of a non-randomised, open-label, phase 2 trial. *The lancet oncology* **2016**, *17* (7), 976-983.
(128) Wilgenhof, S.; Corthals, J.; Heirman, C.; van Baren, N.; Lucas, S.; Kvistborg, P.; Thielemans, K.; Neyns, B. Phase II study of autologous monocyte-derived mRNA electroporated dendritic cells (TriMixDC-MEL) plus ipilimumab in patients with pretreated advanced melanoma. *J clin oncol* **2016**, *34* (12), 1330-1338.

(129) Weber, J.; Gibney, G.; Kudchadkar, R.; Yu, B.; Cheng, P.; Martinez, A. J.; Kroeger, J.; Richards, A.; McCormick, L.; Moberg, V. Phase I/II study of metastatic melanoma patients treated with nivolumab who had progressed after ipilimumab. *Cancer immunology research* **2016**, *4* (4), 345-353.
(130) Merchant, M. S.; Wright, M.; Baird, K.; Wexler, L. H.; Rodriguez-Galindo, C.; Bernstein, D.; Delbrook, C.; Lodish, M.; Bishop, R.; Wolchok, J. D. Phase I clinical trial of ipilimumab in pediatric patients with advanced solid tumors. *Clinical Cancer Research* **2016**, *22* (6), 1364-1370.
(131) Freeman-Keller, M.; Kim, Y.; Cronin, H.; Richards, A.; Gibney, G.; Weber, J. S. Nivolumab in resected and unresectable metastatic melanoma: characteristics of immune-related adverse events and association with outcomes. *Clinical Cancer Research* **2016**, *22* (4), 886-894.
(132) Hamanishi, J.; Mandai, M.; Ikeda, T.; Minami, M.; Kawaguchi, A.; Murayama, T.; Kanai, M.; Mori, Y.; Matsumoto, S.; Chikuma, S. Safety and antitumor activity of anti–PD-1 antibody, nivolumab, in patients with platinum-resistant ovarian cancer. *Journal of Clinical Oncology* **2015**, *33* (34), 4015-4022.
(133) Horinouchi, H.; Yamamoto, N.; Fujiwara, Y.; Sekine, I.; Nokihara, H.; Kubota, K.; Kanda, S.; Yagishita, S.; Wakui, H.; Kitazono, S. Phase I study of ipilimumab in phased combination with paclitaxel and carboplatin in Japanese patients with non-small-cell lung cancer. *Investigational new drugs* **2015**, *33*, 881-889.
(134) Calabrò, L.; Morra, A.; Fonsatti, E.; Cutaia, O.; Fazio, C.; Annesi, D.; Lenoci, M.; Amato, G.; Danielli, R.; Altomonte, M. Efficacy and safety of an intensified schedule of tremelimumab for chemotherapy-resistant malignant mesothelioma: an open-label, single-arm, phase 2 study. *The Lancet Respiratory Medicine* **2015**, *3* (4), 301-309.
(135) Hodi, F.; Lawrence, D.; Lezcano, C.; Wu, X.; Zhou, J.; Sasada, T.; Zeng, W.; Giobbie-Hurder, A.; Atkins, M.; Ibrahim, N. *Bevacizumab plus ipilimumab in patients with metastatic melanoma. Cancer Immunol. Res. 2014; 2: 632–642. doi: 10.1158/2326-6066*; CIR-14-0053.[Europe PMC free article][Abstract][CrossRef][Google Scholar].
(136) Weber, J. S.; Kudchadkar, R. R.; Yu, B.; Gallenstein, D.; Horak, C. E.; Inzunza, H. D.; Zhao, X.; Martinez, A. J.; Wang, W.; Gibney, G. Safety, efficacy, and biomarkers of nivolumab with vaccine in ipilimumab-refractory or-naive melanoma. *Journal of clinical oncology* **2013**, *31* (34), 4311.
(137) Calabrò, L.; Morra, A.; Fonsatti, E.; Cutaia, O.; Amato, G.; Giannarelli, D.; Di Giacomo, A. M.; Danielli, R.; Altomonte, M.; Mutti, L. Tremelimumab for patients with chemotherapy-resistant advanced malignant mesothelioma: an open-label, single-arm, phase 2 trial. *The Lancet Oncology* **2013**, *14* (11), 1104-1111.
(138) Le, D. T.; Lutz, E.; Uram, J. N.; Sugar, E. A.; Onners, B.; Solt, S.; Zheng, L.; Diaz Jr, L. A.; Donehower, R. C.; Jaffee, E. M. Evaluation of ipilimumab in combination with allogeneic pancreatic tumor cells transfected with a GM-CSF gene in previously treated pancreatic cancer. *Journal of immunotherapy (Hagerstown, Md.: 1997)* **2013**, *36* (7), 382.
(139) McNeel, D. G.; Smith, H. A.; Eickhoff, J. C.; Lang, J. M.; Staab, M. J.; Wilding, G.; Liu, G. Phase I trial of tremelimumab in combination with short-term androgen deprivation in patients with PSA-recurrent prostate cancer. *Cancer Immunology, Immunotherapy* **2012**, *61*, 1137-1147.
(140) Madan, R. A.; Mohebtash, M.; Arlen, P. M.; Vergati, M.; Rauckhorst, M.; Steinberg, S. M.; Tsang, K. Y.; Poole, D. J.; Parnes, H. L.; Wright, J. J. Ipilimumab and a poxviral vaccine targeting prostate-specific antigen in metastatic castration-resistant prostate cancer: a phase 1 dose-escalation trial. *The lancet oncology* **2012**, *13* (5), 501-508.
(141) Di Giacomo, A. M.; Danielli, R.; Calabrò, L.; Bertocci, E.; Nannicini, C.; Giannarelli, D.; Balestrazzi, A.; Vigni, F.; Riversi, V.; Miracco, C. Ipilimumab experience in heavily pretreated patients with melanoma in an expanded access program at the University Hospital of Siena (Italy). *Cancer immunology, immunotherapy* **2011**, *60*, 467-477.
(142) Royal, R. E.; Levy, C.; Turner, K.; Mathur, A.; Hughes, M.; Kammula, U. S.; Sherry, R. M.; Topalian, S. L.; Yang, J. C.; Lowy, I. Phase 2 trial of single agent Ipilimumab (anti-CTLA-4) for locally advanced or metastatic pancreatic adenocarcinoma. *Journal of immunotherapy (Hagerstown, Md.: 1997)* **2010**, *33* (8), 828.
(143) Ralph, C.; Elkord, E.; Burt, D. J.; O'Dwyer, J. F.; Austin, E. B.; Stern, P. L.; Hawkins, R. E.; Thistlethwaite, F. C. Modulation of lymphocyte regulation for cancer therapy: a phase II trial of tremelimumab in advanced gastric and esophageal adenocarcinoma. *Clinical Cancer Research* **2010**, *16* (5), 1662-1672.
(144) Yang, J. C.; Hughes, M.; Kammula, U.; Royal, R.; Sherry, R. M.; Topalian, S. L.; Suri, K. B.; Levy, C.; Allen, T.; Mavroukakis, S. Ipilimumab (anti-CTLA4 antibody) causes regression of metastatic renal cell cancer associated with enteritis and hypophysitis. *Journal of immunotherapy (Hagerstown, Md.: 1997)* **2007**, *30* (8), 825.

(145) Maker, A. V.; Yang, J. C.; Sherry, R. M.; Topalian, S. L.; Kammula, U. S.; Royal, R. E.; Hughes, M.; Yellin, M. J.; Haworth, L. R.; Levy, C. Intrapatient dose escalation of anti–CTLA-4 antibody in patients with metastatic melanoma. *Journal of immunotherapy (Hagerstown, Md.: 1997)* **2006**, *29* (4), 455.
(146) Reuben, J. M.; Lee, B. N.; Li, C.; Gomez-Navarro, J.; Bozon, V. A.; Parker, C. A.; Hernandez, I. M.; Gutierrez, C.; Lopez-Berestein, G.; Camacho, L. H. Biologic and immunomodulatory events after CTLA-4 blockade with ticilimumab in patients with advanced malignant melanoma. *Cancer* **2006**, *106* (11), 2437-2444.
(147) Maker, A. V.; Phan, G. Q.; Attia, P.; Yang, J. C.; Sherry, R. M.; Topalian, S. L.; Kammula, U. S.; Royal, R. E.; Haworth, L. R.; Levy, C. Tumor regression and autoimmunity in patients treated with cytotoxic T lymphocyte–associated antigen 4 blockade and interleukin 2: a phase I/II study. *Annals of surgical oncology* **2005**, *12*, 1005-1016.
